# Guiding blind signal separation in X-ray absorption spectroscopy using supervised machine learning

**DOI:** 10.1039/d6sc03428d

**Published:** 2026-07-23

**Authors:** Janis Timoshenko, Andrea Martini, Martina Rüscher, Lichen Bai, Leonardo Domenico De Angelis, Mariana Cecilio de Oliveira Monteiro, Beatriz Roldan Cuenya

**Affiliations:** a Department of Interface Science, Fritz-Haber Institute of the Max-Planck Society Berlin 14195 Germany janis@fhi-berlin.mpg.de roldan@fhi-berlin.mpg.de; b Instituto de Química, Universidade de São Paulo Av. Prof. Lineu Prestes 748 05508-900 São Paulo SP Brazil

## Abstract

Transformations under working conditions experienced by heterogeneous catalysts and other functional materials commonly result in complex, time-dependent atomistic structures, where the active species that are relevant for the functionality of the material coexist with passive spectators. Decoupling the contributions of multiple coexisting species to spectroscopic data remains a common challenge for many sample-averaging techniques, including X-ray absorption spectroscopy. Here we demonstrate that a data-driven, supervised machine learning based approach can greatly assist in the challenging task of singling-out the contributions of spectroscopically distinct species. This is accomplished by recognizing common patterns in large datasets of X-ray absorption spectra that are calculated *ab initio* for a broad range of different compounds. Furthermore, our approach also serves as a convenient tool for the quantification of the uncertainties of spectral decomposition. We demonstrate the application of our method to model datasets and also to experimental spectra from time-resolved *operando* X-ray absorption measurements. The latter are carried out for a copper oxide-derived catalyst during electrocatalytic CO_2_ conversion in an oxalic acid-based electrolyte. Our method reveals an intriguing charge disproportionation process, resulting in the formation of copper oxalate species and metallic Cu already under open circuit conditions.

## Abstract 2

Here supervised machine learning guides the disentanglement of contributions of multiple coexisting species to the sample-averaged spectroscopic data, by recognizing common patterns in large datasets of spectra for a broad range of compounds.

## Graphical abstract

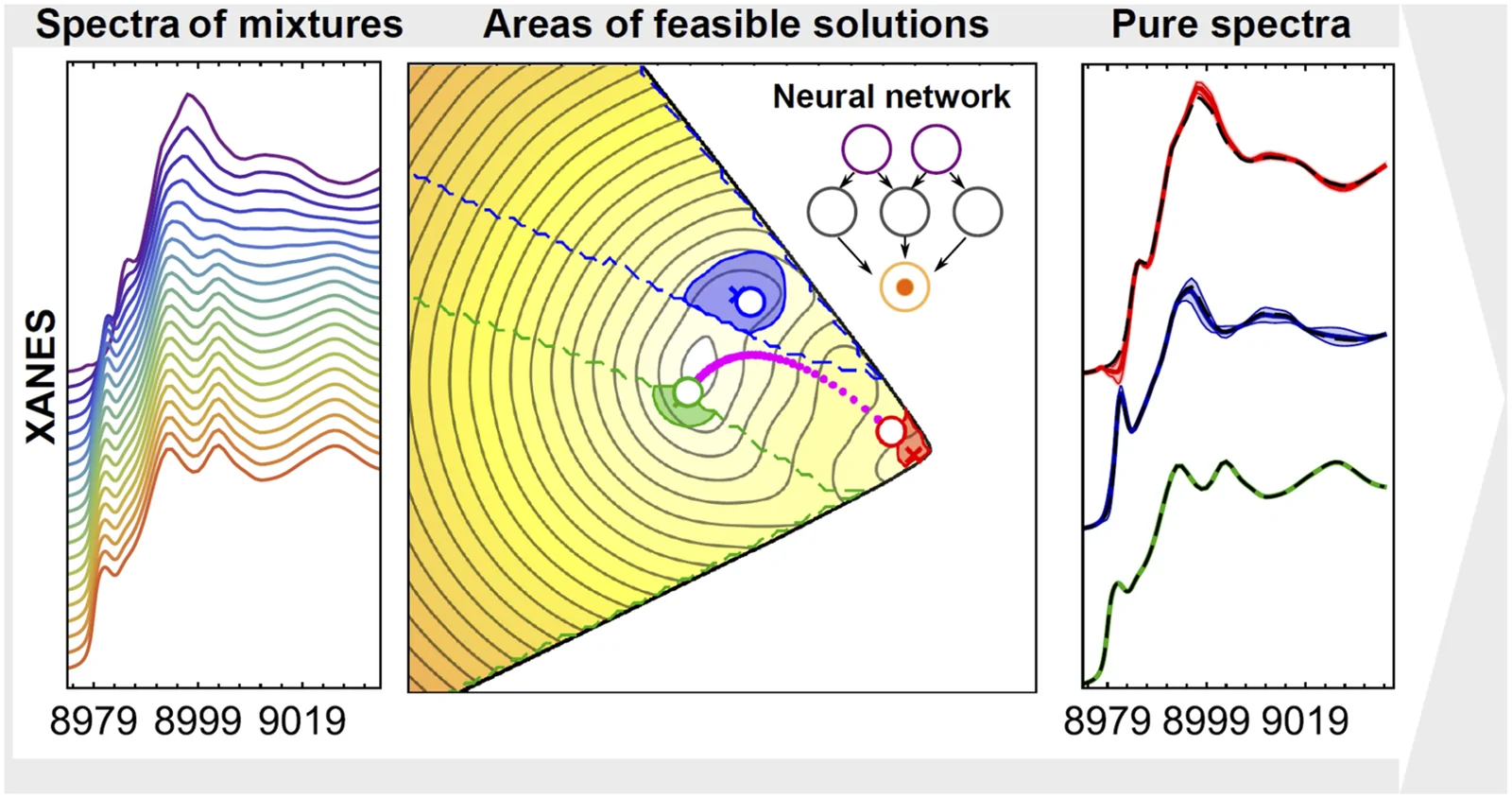

## 1. Introduction

The common challenge for many spectroscopic techniques is disentangling the contributions of distinct species that coexist in the analysed sample and contribute simultaneously to the sample-averaged spectra. In signal processing, this problem is known as a “blind source (signal) separation” or “cocktail party” problem, while in spectroscopy it is commonly referred to as a “multivariate curve resolution” (MCR) problem.^1,2^ For spectroscopies where the Beer–Lambert law is applicable,^2^ the spectra of mixtures can be expressed as linear combinations of spectra of pure species, mixed with some weights, which are linked to the relative concentrations of the pure species. In general, neither the pure spectra, nor their weights are known. However, several spectra of different mixtures with different weights of pure components can be collected (*e.g.*, by performing measurements at various stages of a chemical reaction or in different spots of a non-homogeneous sample). Furthermore, one often can apply prior knowledge about the possible shapes of the pure spectra and the values of their corresponding concentrations. This allows one to narrow down the viable solutions to the MCR problem. Starting from the seminal work of Lewton and Sylvestre,^3^ different methods have been proposed for this purpose, such as iterative transform target factor analysis (ITTFA),^4,5^ multivariate curve resolution with alternating least squares (MCR-ALS),^6–10^ and the transformation matrix (TM) approach.^11–14^ However, it is important to stress that the results provided by these methods are almost always ambiguous, since many vastly different solutions can satisfy the imposed constraints.^15^ The outcome is thus strongly biased by the algorithm initialization (for iterative methods like MCR-ALS and ITTFA) and the imposed explicit or implicit additional assumptions. The success of these methods thus widely varies from one case to another, and, in general, the MCR problem remains unsolved.

Here, we address this issue by employing supervised machine learning (SML) for guiding the selection of the solution to the MCR problem. We apply this method for the interpretation of X-ray absorption near edge structure (XANES) spectra, although it can be used for other spectroscopies as well. The MCR problem features prominently in XANES analysis, since *operando* X-ray absorption spectroscopy (XAS) is often used for tracking the time-dependent evolution of the samples with different coexisting species.^16^ The conventional approach for the interpretation of XANES spectra of heterogeneous samples – linear combination fitting (LCF) – requires *a priori* knowledge of the reference spectra corresponding to individual pure species that are believed to be present. It is thus often inapplicable to studies of working functional materials, where unique, transient species can be present with no analogues among the standard reference materials. For this reason, MCR methods were introduced to XAS spectroscopy already a few decades ago,^17–19^ and their application is getting increasingly popular. As a parallel and more recent development, SML has been introduced for the quantitative interpretation of XANES spectra.^20–26^ However, so far, the applicability of SML methods has been limited to the spectra of pure species. The few existing efforts dealing with the coupling of MCR with SML are mostly based on the use of conventional MCR tools to obtain the approximations of the pure spectra, and then applying SML for their interpretation.^27–30^ Furthermore, several studies tried to couple SML-assisted XANES fitting with the refinement of concentration profiles.^14,31^ However, these approaches by themselves are not able to address the ambiguity issue of MCR.

In our approach, we first train an SML routine to distinguish physically reasonable XANES spectra from the unphysical ones by training an artificial neural network (ANN) on a large set of mixtures of theoretically calculated XANES spectra for different pure structures extracted from the Materials Project database.^32–34^ Next, for a given series of XANES spectra corresponding to mixtures of unknown pure species, we identify all the solutions to the MCR problem, which are consistent with certain imposed constraints, such as non-negativity of spectra and concentration profiles. At the last step, from the obtained solutions we choose one that results in the most physically reasonable spectra for pure species, as judged by our ANN. In this study, we demonstrate the feasibility of this approach using both theoretical and experimental data. We also assess the sensitivity limits of the proposed method, which can be helpful for understanding the sensitivity limits of MCR approaches in general. Furthermore, we apply this approach for the interpretation of experimental time-resolved *operando* XANES data collected for Cu-based electrocatalysts evolving under electrocatalytic CO_2_ reduction reaction (CO_2_RR) conditions in an oxalic acid-based electrolyte. Our method allows us to track the charge disproportionation and reduction processes, providing the first *operando* insight into the evolution of the Cu active states in this electrocatalytic system.

## 2. Experimental and simulations

### 2.1 Experimental measurements

Cu_2_O nanocubes (Cu NCs) were prepared following the wet-chemical synthesis procedure as reported before.^35^ Time-resolved *operando* XAS spectra were collected at the ROCK beamline at the SOLEIL synchrotron radiation facility (Sant Aubin, France). The measurements were performed in fluorescence mode at the Cu K-edge (8979 eV), using a PIPS detector. A channel-cut Si(111) monochromator continuously oscillating at 1 Hz frequency was used for energy selection, resulting in a time resolution of up to 0.5 s per spectrum. XAS measurements for the reference samples were carried out in transmission mode. The intensity of incident X-rays before and after the sample was monitored with an ionization chamber filled with pure N_2_.

*Operando* XAS measurements were performed in our in-house built single compartment electrochemical cell. The sample was spray-coated onto a carbon-based gas diffusion electrode (GDE), which was taped on the cell body with Kapton tape, acting both as a working electrode and as a window for incident and fluorescent X-rays. A Pt mesh was used as a counter-electrode, and a reversible hydrogen electrode (RHE) was used as a reference electrode. As electrolyte, we used a CO_2_-saturated mixture of 0.12 M H_2_C_2_O_4_ and 0.10 M KOH (bulk pH = 1.9) which was constantly circulated in the electrochemical cell, with CO_2_ continuously bubbling through the electrolyte. Measurements were first performed in air, followed by 15 min of measurements at open circuit potential (OCP) in electrolyte. Next, a constant current of −53 mA (corresponding to −120 mA cm^−2^ current density) was applied for 1 hour. The applied current was controlled using a BioLogic potentiostat (SP300), equipped with a booster. Finally, the catalyst was measured again under OCP conditions for additional 15 min.

Calibration and averaging of collected XAS data were first performed using the dedicated software at the beamline, where we used the known energies of monochromator glitches for the alignment of the energy scale. Energy values corresponding to glitches were determined based on XAS measurements for Cu foil. Furthermore, data re-binning, merging, normalization and extraction of the XANES spectra were performed using a series of in-house built *Wolfram Mathematica*^36^ scripts. Principal component analysis and the SML-guided reconstruction of the pure spectra were similarly performed using in-house built *Wolfram Mathematica* scripts.

Extended X-ray absorption fine structure (EXAFS) data were extracted using a script employing LARCH code.^37^ EXAFS fitting was performed using FEFFIT code^38^ for Cu–O and Cu–Cu scattering paths. Cu–O and Cu–Cu coordination numbers (CNs), interatomic distances and disorder factors (Debye–Waller factors *σ*^2^) were fitted, as well as the correction to the photoelectron reference energy (Δ*E*_0_). The amplitude reduction factors (*S*_0_^2^ factors) for Cu–Cu and Cu–O bonds were obtained from the fit of reference EXAFS spectra of Cu foil and Cu_2_O and were 0.90 and 0.73, respectively. Fitting was performed in *R*-space between 1.0 and 2.8 Å. This range was reduced to 1.92 Å for the fully oxidized catalyst and references. Fourier transform was carried out in the *k*-range between 3.0 and 9.5 Å^−1^.

Reference spectra of Cu foil, Cu_70_Zn_30_ foil, Cu_1_Ag_25_ powder, Cu_2_O, CuI, CuO, Cu(OH)_2_, Cu(CH_3_COO)_2_, CuCO_3_·Cu(OH)_2_, CuSO_4_, CuCl_2_, CuClO_4_ and CuPc (copper(ii) phthalocyanine) were collected during the same beamtime at SOLEIL ROCK. A reference spectrum of Cu_3_N was taken from our previous work.^39^ Reference spectra of Cu_2_S, CuS and Cu_3_(CO_3_)_2_(OH)_2_ (azurite) were taken from the XASLIB database (https://xaslib.xrayabsorption.org/).^40^ Reference spectra of CuNi, CuCl, CuBr_2_, CuF_2_, CuCr_2_O_4_, CuMoO_4_, CuSe, CuWO_4_, Cu(acac)_2_ (Cu(ii) acetylacetonate) and CuC_2_O_4_·H_2_O (Cu(ii) oxalate) were taken from the MDR XAS database (https://mdr.nims.go.jp/).^41^

### 2.2 *Ab initio* XANES simulations

Cu K-edge XANES simulations were carried out using the FDMNES code^42^ for the Cu-containing crystal structures downloaded from the Materials Project database.^32^ In addition, calculations for several compounds (CuPc, Cu(acac)_2_, and Cu_3_(CO_3_)_2_(OH)_2_) that are not included in the Materials Project were also carried out using their known structures.^43–45^ Furthermore, calculations for fcc-type alloys (Cu_70_Zn_30_, CuNi, and Cu_1_Ag_25_) were performed starting from the 4 × 4 × 4 supercell of pure Cu, by randomly replacing the necessary number of atoms with secondary metal atoms to achieve the target alloy composition, and rescaling the lattice constants according to Vegard's law and averaging the spectra calculated for non-equivalent Cu sites.

For most calculations, we used the FDMNES version 29 (from February 2024). Simulations were carried out using muffin-tin-approximation. While the finite difference (FD) approach, also implemented in FDMNES, is believed to be in some cases more accurate, we have found that for our purposes the muffin-tin approximation provided a good compromise between computational resources required and the accuracy of calculated spectra. However, for some structures, featuring heavy atoms (CuI, CuPbO_3_, Cu_4_I_3_Cl, and CuPb_3_), these calculations with the latest FDMNES version resulted in strongly shifted spectra. For these materials, calculations were repeated using the FDMNES version 15 (from February 2019) and employing the FD approach. The cluster size for the FDMNES calculations was set to 7 Å. Further increase in the cluster size did not change the main spectroscopic features significantly. All other calculation parameters were set to their default values, including the values for convolution parameters, as implemented in the FDMNES code. In total, 3679 FDMNES calculations have been carried out, making it one of the world-wide largest collections of FDMNES-calculated Cu K-edge XANES spectra. All calculated spectra and the corresponding FDMNES input files are available in the SI.

We emphasize that the critical point in making the simulated spectra useful for the interpretation of the experimental data is to ensure the reliable alignment of the simulated spectra with each other and with the experimental data. To address this issue, for the spectra alignment here we relied on a semi-supervised machine learning approach.^46–48^ Here, we first aligned the theoretically calculated spectra for which we had corresponding experimental data available either from our own experimental measurements for the reference samples or from the XAFS databases.^41,49^ These 24 aligned spectra were used to establish the relationship between the spectra and the necessary energy shift, which was then interpolated on the remaining calculated spectra. We discuss this approach in detail in Note S1 and Fig. S1 in the SI. In total, after removing the spectra that could not be reliably aligned, we ended up with 1994 calculated Cu K-edge XANES spectra for materials with a broad range of different structural motifs, alloy compositions and different Cu chemical states.

## 3. Results and discussion

### 3.1 Areas of feasible solutions

Let us consider a set of *m* normalized experimental XANES spectra *µ*_*i*_(*E*) that track the changes in the sample speciation. We then assume that the differences between these spectra can be assigned solely to the changes in the concentrations of the constituent species. Thus, the *µ*_*i*_(*E*) can be expressed as linear combinations of *n* spectra for pure species *s*_*j*_(*E*):1
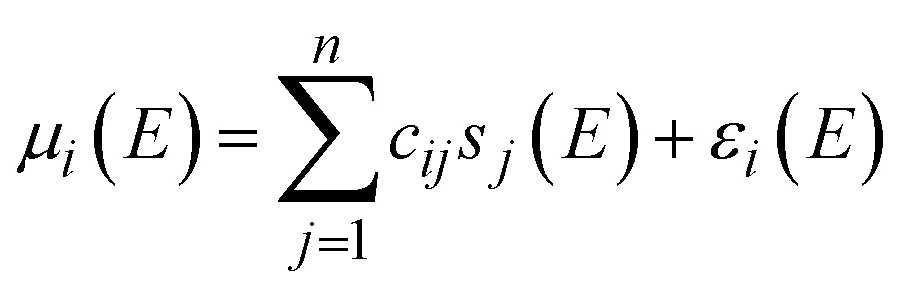
where *c*_*ij*_ encodes the concentration of the *j*-th species in the *i*-th XANES spectrum and *ε*_*i*_(*E*) represents the experimental noise. In matrix form, we rewrite this as follows:

2 ***M*** = ***CS*** + ***E***

With each spectrum containing *k* datapoints, ***M*** is an *m* × *k* matrix, with each row corresponding to a single measured spectrum; ***C*** is an *m* × *n* matrix, with each row containing the concentrations of the *n* pure species; and ***S*** is an *n* × *k* matrix, containing the spectra of the pure species. In a general case, neither ***C*** nor ***S*** are known. Matrix ***E*** describes the contribution of the experimental noise.

By principal component analysis (PCA),^50–52^ we express the rows in ***M*** as linear combinations of a few orthogonal vectors referred to as principal components (PCs). The minimal number of such vectors required to describe the data determines the dimensionality (rank) of ***M*** and (if the experimental noise can be neglected) is equal to the number of pure species. Here, we assume that (i) all pure species are spectroscopically distinct; (ii) there are no species whose concentrations do not change at all between different measurements; and (iii) the concentration profiles of different species are linearly independent. PCs can be obtained using standard factorization techniques,^53^ such as singular value decomposition (SVD), by decomposing ***M*** as follows:

3 ***M*** = ***UΣV***

***U*** and ***V*** are two square matrices with dimensions (*m* × *m*) and (*k* × *k*), respectively; ***Σ*** is a (*m* × *k*) rectangular matrix and has non-zero elements on its diagonal only. These elements (singular values) are related to the eigenvalues of the ***M***^*T*^***M*** covariance matrix. PCs are given by the corresponding eigenvectors *v*_*j*_ and constitute the rows of ***V***. ***UΣ*** corresponds to the projections of spectra in ***M*** on these PCs.

For the dataset stemming from mixing *n* pure species, only the first *n* PCs are physically meaningful, while the remaining PCs contain just experimental noise. Thus, eqn (3) can be rewritten as follows:4
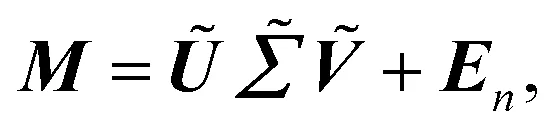
where ***Ṽ*** contains now only the first *n* rows of ***V*** (first *n* PCs), ***Ũ*** contains only the first *n* columns of ***U***, and 
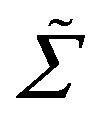
 is the *n* × *n* diagonal matrix containing the first *n* singular values. The ***E***_*n*_ matrix describes the error of the reconstruction using *n* PCs.

Eqn (4) resembles strongly eqn (2), with 
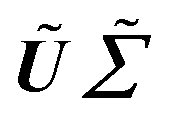
 playing the role of ***C*** and ***Ṽ*** playing the role of *S*. However, the PCs themselves do not correspond to any particular species and each contains a combination of spectroscopic features stemming from different pure components. Nonetheless, the spectra of pure species can be represented as linear combinations of these PCs, since the PCs form an orthogonal set of basis vectors that spans ***M***. Following the approach by Lawton and Sylvestre,^3^ we write5
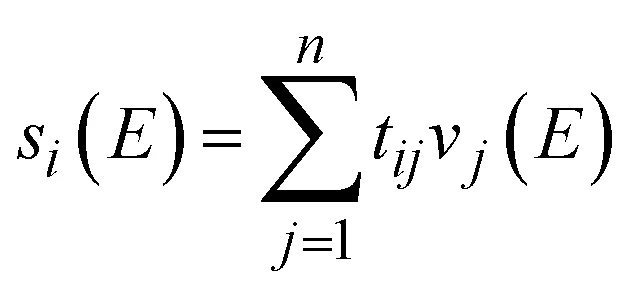
or in matrix form,

6 ***S*** = ***TṼ***

The concentration profiles can be written as follows:7
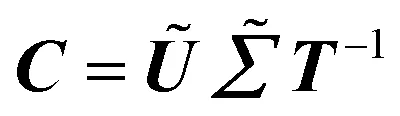


Any values of the *t*_*ij*_ coefficients constituting an *n* × *n* dimensional transformation matrix *T* will result in a set of “spectra” *s*_*i*_(*E*) that would formally solve eqn (2). However, only a few of these *t*_*ij*_ values will result in spectra and concentration profiles that satisfy the expected non-negativity and normalization conditions for XANES spectra and concentration profiles. We can represent each of the combination of *t*_*ij*_ values as a point in *n* × *n*-dimensional space. The subspace in this *n* × *n*-dimensional space that results in a solution to the spectral decomposition problem and satisfies all the imposed constraints is referred to as “area of feasible solutions” (AFS).^15,27,54–56^

First, to fix the values of the *t*_*i*1_ coefficients, we note that the first PC plays a special role in the PCA of XANES spectra. While this is not true for arbitrary signals, for XANES, where the variations between different spectra are much lower than the magnitudes of the spectra themselves, the first PC is close (up to a constant multiplier) to the average spectrum, calculated over the entire dataset ***M***. Furthermore, we note that the contribution of this first PC to all spectra in the dataset ***M*** is almost the same. This is a consequence of the fact that the spectra are normalized, and the concentrations of pure species add to 1 (mass balance condition).^15^ To what accuracy this property is satisfied depends somewhat on the algorithm that is used for spectra normalization. To avoid this, it is convenient to renormalize the analysed spectra by dividing them by their Euclidean norms 
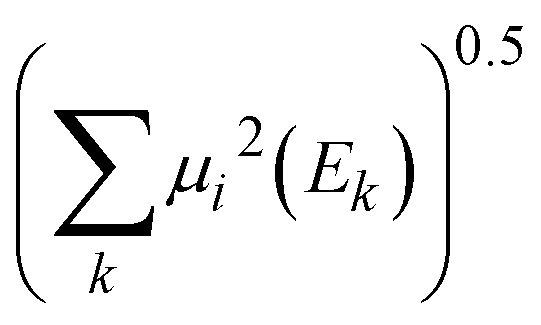
. The summation here is carried out over all the energy points within the energy range that is used for analysis. Under these conventions, the projections of all spectra in the analysed dataset on the first PC become very close to 1. Crucially, the requirement that the spectra of pure species are normalized, and that their concentrations add to 1 at all times, can now be easily satisfied, just by setting to 1 all the projections of pure spectra on the first PC (*i.e.*, all *t*_*i*1_ in eqn (5) must be equal to 1). We can thus represent any spectrum in our dataset, as well as the unknown spectra of pure species, as points in (*n* − 1)-dimensional space, where the coordinates correspond to the projections of these spectra on the 2nd, 3rd, *etc.*, PCs.

To further constrain other elements of the transformation matrix, we use the condition that the XANES spectra of pure species must be non-negative. Since the 1st PC is equal to the average spectrum, the first PC is always non-negative. Furthermore, since all PCs are constructed to be orthogonal to each other, this automatically means that all other PCs are oscillating around 0. As a result, the absolute value of *t*_*ij*_ cannot be too large: if any *t*_*ij*_ is too negative, then its product *t*_*ij*_*v*_*j*_(*E*) with the portion of the PC *v*_*j*_(*E*) that is larger than 0 will become so negative that the overall sum in eqn (5) will be also negative, violating the non-negativity constraint. If *t*_*ij*_ is positive and too large, the same argument applies to the negative portion of the PC. The range of possible *t*_*ij*_ values is always limited. For all pure species, the allowed *t*_*ij*_ values (*j* > 1) will thus form a convex polyhedron in (*n* − 1) dimensional space, which we refer to as a “spectra-specific” AFS. Note that since the spectra of all pure species are constructed from the same PCs and are subject to the same constraints, for a given dataset ***M*** the “spectra-specific” AFS is the same for all pure species. An example of such “spectra-specific” AFS for a mixture of three species is given in Fig. 1a and b. Any candidate solution to the MCR problem can now be constructed by combining *n* points from the (*n* − 1)-dimensional spectra-specific AFS. However, one still needs to ensure that the concentration profiles corresponding to such set of points are non-negative. The latter condition has a simple geometrical interpretation. Let us consider all the points <svg xmlns="http://www.w3.org/2000/svg" version="1.0" width="17.272727pt" height="16.000000pt" viewBox="0 0 17.272727 16.000000" preserveAspectRatio="xMidYMid meet"><metadata> Created by potrace 1.16, written by Peter Selinger 2001-2019 </metadata><g transform="translate(1.000000,15.000000) scale(0.015909,-0.015909)" fill="currentColor" stroke="none"><path d="M560 840 l0 -40 -160 0 -160 0 0 -40 0 -40 160 0 160 0 0 -40 0 -40 40 0 40 0 0 40 0 40 40 0 40 0 0 40 0 40 -40 0 -40 0 0 40 0 40 -40 0 -40 0 0 -40z M240 520 l0 -40 -40 0 -40 0 0 -40 0 -40 40 0 40 0 0 40 0 40 40 0 40 0 0 -160 0 -160 -40 0 -40 0 0 -40 0 -40 -40 0 -40 0 0 -40 0 -40 80 0 80 0 0 40 0 40 40 0 40 0 0 200 0 200 80 0 80 0 0 -40 0 -40 -40 0 -40 0 0 -40 0 -40 40 0 40 0 0 -40 0 -40 -40 0 -40 0 0 -120 0 -120 120 0 120 0 0 40 0 40 40 0 40 0 0 40 0 40 -40 0 -40 0 0 -40 0 -40 -40 0 -40 0 0 40 0 40 -40 0 -40 0 0 40 0 40 40 0 40 0 0 120 0 120 80 0 80 0 0 40 0 40 -280 0 -280 0 0 -40z"/></g></svg> 
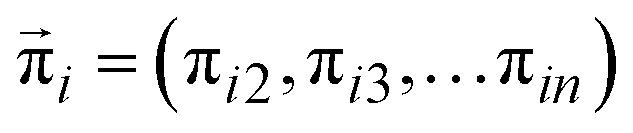
 corresponding to the projections of experimental spectra in the original dataset on the PCs. In Fig. 1, these points are indicated with magenta colour. Considering that experimental spectra are non-negative, all these points belong to the spectra-specific AFS. Now, the selected *n* points from the spectra-specific AFS form a valid set of pure spectra that always satisfies the mass-balance condition if and only if these *n* points form a simplex (a triangle for *n* = 2, a tetrahedron for *n* = 3, *etc.*) that encompasses all the 
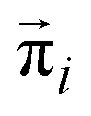
 points. This conclusion follows directly from the properties of the barycentric coordinate system associated with the simplex vertices in (*n* − 1)-dimensional space. For example, the vertices of any such triangle, as shown with black lines in Fig. 1b, would correspond to a “solution” to the MCR problem that satisfies all the imposed constraints. Clearly, many of such solutions correspond to completely unphysical spectra (Fig. 1c). To further narrow down the AFS, additional explicit or implicit assumptions are needed. For example, in iterative methods such as MCR-ALS, the implicit assumption is that the solution (true spectra or concentration profiles) should not be too far away from the initial guess of the spectra of pure species (or initial guess for concentration profiles). This approach works well in the cases where at some points during the experiment all the species appear in a nearly pure state. In fact, Manne *et al.* formulated the so called “resolution theorems” that state that the MCR problem can be solved unambiguously without additional information if for each pure species in the analysed dataset there exists a sufficient number of different mixture spectra to which this pure species does not contribute.^57^ In this ideal case, the solution to the MCR problem can be obtained without the need for any iterations, simply by choosing the smallest possible one from the aforementioned set of valid simplices.^58^ However, such complete conversion of species is not always observed.

**Fig. 1:**
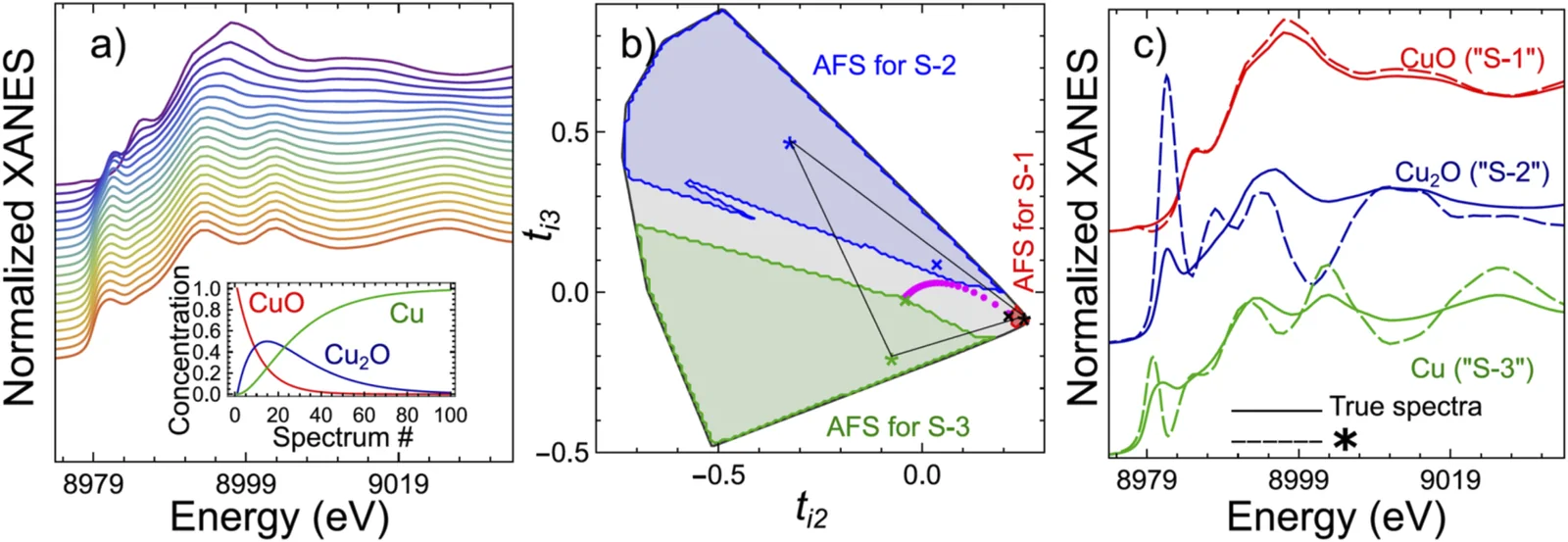
Construction of AFS for the model dataset. (a) Model dataset consisting of 100 Cu K-edge XANES spectra, constructed as linear combinations of metallic Cu, Cu_2_O and CuO reference spectra, using the concentration profiles shown in the inset. (b) Spectra-specific AFS (grey polygon), indicating the range of *t*_*ij*_ coefficients resulting in non-negative linear combinations of the first 3 PCs, extracted from the dataset shown in (a). Here, the border of spectra-specific AFS is constructed numerically point-by-point, by following the lines starting from the (0,0) point (which corresponds to the average spectrum and, thus, is always located within the spectra-specific AFS) and employing the bisection method to find the point along each line where 
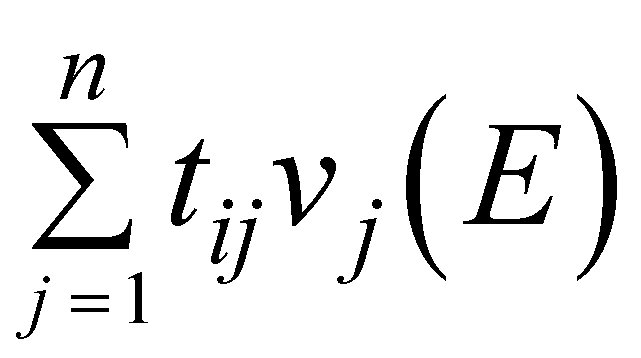
 becomes equal to 0. Magenta points correspond to the projections of the spectra in (a) on the extracted PCs. Black, blue and green crosses correspond to the true solution to the MCR problem, namely, to the projections of reference spectra of CuO, Cu_2_O and Cu on the extracted PCs. Red, blue and green areas correspond to the ranges of viable solutions for the first, second and third pure species (S-1, S-2 and S-3, respectively) that also satisfy the non-negativity of the concentration profiles. Any simplex with vertices in red, green and blue regions that encompasses all magenta points will formally result in a valid solution to the MCR problem. An example of such a simplex (triangle) is shown in (b) with black lines and black, green and blue stars as vertices. Corresponding spectra are shown with dashed lines and are compared with the true solution in (c). The red, green and blue regions of feasible solutions here are obtained by the brute-force method, by sampling each combination of 3 points from the discretized spectra-specific AFS, and checking whether all the magenta points are located within the obtained 3-point simplex. Note that depending on the spectroscopic contrast between different pure species and the specific concentration profiles, such clear separation of the AFS regions corresponding to S-1, S-2 and S-3 species is not always possible, and, in general case, red, blue and green regions can overlap. Nonetheless, this affects only the visualization of the space of possible solutions and does not affect the algorithm for finding the optimal solution to the MCR problem as discussed below.

However, based on the shape of the obtained spectral features in the candidate solutions for spectra of pure species, an experienced researcher can often distinguish the realistic ones from the unphysical ones. This approach is referred to as the “transformation matrix approach”.^15,27^ For example, in the PyFitIt code^59^ the elements of the transformation matrix *t*_*ij*_ are tuned manually by moving the corresponding sliders in a graphical user interface, until “physically reasonable” spectra and concentration profiles are obtained. It is clear, however, that such an approach is highly biased by the user's previous experience.

Considering the recent successful applications of machine learning methods in the classification of XANES spectra,^20,60,61^ an attractive approach for addressing this issue would be designing an SML routine that would be able to distinguish between realistic and unfeasible candidate solutions to the spectral decomposition problem, thus reducing the size of the AFS and, correspondingly, the ambiguity in the MCR. The development and validation of such an approach will be discussed in the next sections.

### 3.2 Data-driven narrowing of AFS

To train an SML algorithm for distinguishing between physically plausible and unrealistic XANES spectra, one would need to provide a large number of representative XANES spectra corresponding to a broad range of different materials. The development of databases of experimental XANES spectra is an ongoing effort, and the sizes and quality of these datasets have improved noticeably during the last few years.^40,41,62^ However, for each absorption edge, these databases still contain just a few dozens (in the best case) of unique spectra, which is still insufficient for training a robust machine learning algorithm. However, the last few decades have witnessed a dramatic increase in the accuracy and availability of *ab initio* XANES simulations. As we and others have demonstrated,^20,25,33,34,60,61,63,64^ the quality of theoretically simulated spectra, in many cases, is sufficient to replace the missing experimental data and is a very promising tool for training advanced machine learning algorithms.

Indeed, as shown in Fig. 2, computationally relatively cheap FDMNES simulations are able to capture the main features of experimental Cu K-edge XANES spectra remarkably well and allow one to quickly generate large sets of realistic-looking XANES spectra. We highlight here that the availability of large databases of materials structures such as the Materials Project^32^ is the key to obtaining highly representative datasets featuring metal sites with different coordination and chemical states. We again emphasize that one of the key challenges in employing *ab initio* simulated XANES spectra for the interpretation of experimental data lies in the need to reliably align the simulated spectra with experimental ones. In this work we have addressed this issue *via* an auxiliary semi-supervised ML approach, as discussed in Note S1 and Fig. S1.

**Fig. 2:**
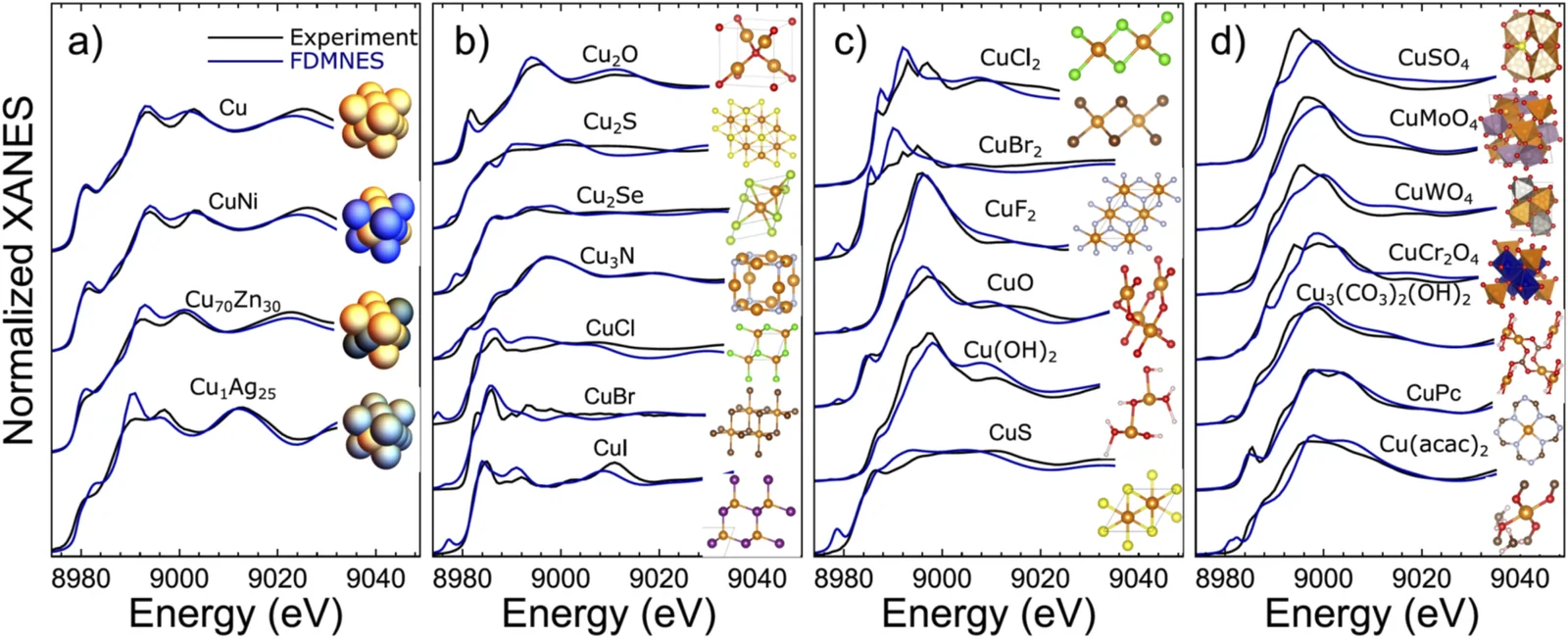
Comparison of experimental and simulated Cu K-edge XANES spectra of the 24 reference compounds. Experimental spectra (black lines) are compared with the results of FDMNES simulations (blue lines) after the alignment. Representative schematics of Cu environments in the different compounds are shown in the inset. Results for metallic Cu compounds (a), monovalent Cu compounds (b) and divalent Cu compounds (c and d) are shown.

The database of simulated and aligned Cu K-edge XANES spectra, featuring a total of 1994 spectra, can provide immediate, highly useful insight for narrowing the space of possible solutions to the MCR problem, even without any further (complicated) algorithm development. Let us return to our previous example (Fig. 1). We can project all 1994 calculated FDMNES spectra on the principal components extracted from the example dataset in Fig. 1a. Each calculated spectrum now can be represented as a point with coordinates (*f*_*i*2_, *f*_*i*3_), corresponding to the projections of the *i*-th FDMNES spectrum on 2nd and 3rd PC, respectively. We can also similarly project the experimental spectra available from our much smaller library of 24 experimental reference spectra of different Cu compounds, resulting in points (*e*_i2_, *e*_i3_). In Fig. 3, we overlap all these points with the AFS identified in Fig. 1b. It is striking to see that the projections of all calculated and experimental spectra corresponding to very different materials with very different Cu local environments and chemical states are all located in a very small area of our AFS. Specifically, all these points correspond to *t*_*i*2_ values between *ca.* −0.05 and +0.32 and *t*_*i*3_ values between *ca.* −0.15 and +0.12. From this simple result, we can immediately conclude that all the points in our AFS outside this narrow region can be safely excluded from the analysis, since they do not resemble any physically reasonable Cu K-edge XANES spectrum. This narrows down the set of possible solutions to the MCR problem dramatically. In fact, in Fig. 3, only the very narrow ranges of *t*_*i*2_ and *t*_*i*3_ values marked by red, blue and green circles correspond to physically reasonable spectra, while simultaneously satisfying the non-negativity constraint for the concentration profiles. Supported by SML algorithms, we can further improve on this already promising result, as we discuss below.

**Fig. 3:**
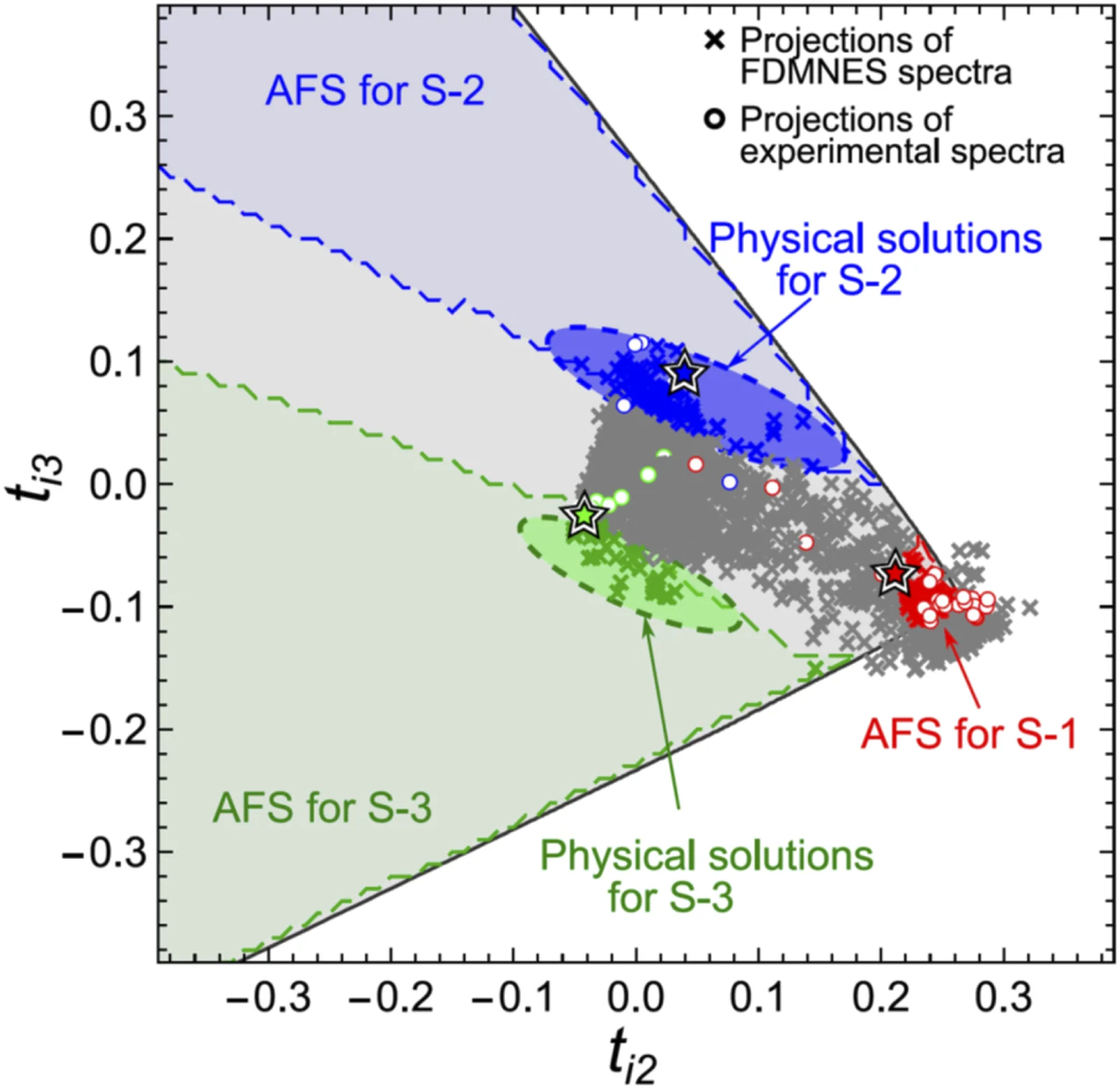
Narrowing the AFS for the model dataset. Green, blue and red stars show the true solution, corresponding to experimental reference spectra of metallic Cu, Cu_2_O and CuO. Crosses show the projections of 1994 simulated and aligned XANES spectra on the PCs extracted from the dataset shown in Fig. 1a. Red, green and blue crosses show the projections of theoretical spectra that fall within the AFS for species S-1, S-2 and S-3, respectively. Gray crosses show the projections of theoretical spectra that do not satisfy the non-negativity constraint for spectra and/or concentration profiles. Circles show projections of 24 experimental spectra on the same PCs. Green, blue and red circles correspond to the experimental spectra of Cu(0), Cu(i) and Cu(ii) species, respectively.

### 3.3 Supervised ML to guide the solution of the MCR problem

From Fig. 3, it is clear that physically reasonable and unphysical spectra are well separable in the latent space of the spectra projections on the PCs extracted from the original dataset ***M***. This means that an SML algorithm should be able to distinguish between these spectra, providing useful guidance and reducing the ambiguity of the MCR solution. One could, *e.g.*, use a simple “nearest-neighbour” approach, where the likeliness of a particular candidate solution is measured by the Euclidean distance from the proposed spectra of pure species to the nearest simulated spectrum in our library. However, we have found that better performance can be achieved by using an artificial neural network approach (ANN-MCR).

In the ANN-MCR method, we construct and train an ANN that takes an XANES spectrum as input and provides as output a single number (score), *ξ*. The score is a measure of how “physically reasonable” the provided spectrum is. We define *ξ* so that for any spectrum of pure species the ANN should return a *ξ* value close to 0, while for spectra that are far from physically reasonable ones, the ANN should return a large *ξ*. The solution to the MCR problem is then obtained by first finding the range of *t*_*ij*_ values that satisfy the imposed constraints and then selecting from this range a single solution that results in the lowest *ξ* values for all pure species spectra.

To train such an ANN, we need to provide a large number of spectra with different *ξ* values. The superior performance of the ANN method is ensured by the fact that during training the ANN is exposed not only to the examples corresponding to low *ξ* values (*i.e.*, physically reasonable pure spectra), but also to spectra with high *ξ* values (*i.e.*, mixtures of different pure spectra). For this purpose, we use our library of FDMNES-generated spectra and the following algorithm:

(1) First, from our generated database of 1994 spectra, we randomly select 70% of the spectra to obtain a smaller subset of FDMNES-simulated spectra.

(2) From this smaller set, we randomly choose two spectra, *µ*^FDMNES^_*i*1_(*E*) and *µ*^FDMNES^_*i*2_(*E*).

(3) We then perform a singular value decomposition of the two-row matrix formed by these two spectra to obtain the corresponding two principal components *v*^T^_*i*1_(*E*) and *v*^T^_*i*2_(*E*).

(4) Next, we generate a training spectrum *µ*^T^_*i*_(*E*) by adding to the first PC the 2nd PC multiplied by a random weight *t*: *µ*^T^_*i*_(*E*) = *v*^T^_*i*1_(*E*) + *tv*^T^_*i*2_(*E*).

(5) The corresponding score *ξ*_*i*_ is then defined as the Euclidean distance between *µ*^T^_*i*_(*E*) and *µ*^FDMNES^_*i*1_(*E*) or *µ*^FDMNES^_*i*2_(*E*), depending on which one of these two spectra is closer to *µ*^T^_*i*_(*E*). *I.e.*,

8 *ξ*_*i*_ = min{‖*µ*^T^_*i*_ − *µ*^FDMNES^_*i*1_‖,‖*µ*^T^_*i*_ − *µ*^FDMNES^_*i*2_‖}

(6) We repeat steps (2)–(5) 100 000 times to generate a training set consisting of 100 000 mixture spectra *µ*^T^_*i*_(*E*) and the corresponding score values *ξ*_*i*_.

(7) Using this training dataset, an artificial neural network is trained.

(8) Steps (1)–(7) are repeated 20 times to obtain 20 different neural networks trained on different training sets. The comparison of the predictions from these 20 ANNs will be used to estimate uncertainties.

Crucially, for the construction of these training spectra, we use only pairwise mixtures of spectra from our library to ensure that all spectra in our original set of calculated spectra have a significant chance to contribute to the final training set. Nonetheless, the ANN trained on this dataset is able to correctly estimate the score *ξ* not only for mixtures of two pure species, but also for mixtures of three, four and more species (Fig. S2). Furthermore, the training can be done using solely theoretical, FDMNES spectra or using a combined dataset consisting of simulated spectra and the available experimental data for standard materials. The former ANNs are referred to as ANN_T_, while the latter ANNs are denoted as ANN_T+E_. For testing purposes, we introduce and train also ANN_T+E-test_, which, similarly to ANN_T+E_, is trained on theoretical and experimental data, but in this case the experimental spectra of Cu foil, Cu_2_O, CuO and Cu(OH)_2_ are not included in the training dataset (since these were the spectra used to construct the model dataset introduced in Fig. 1). Finally, one can even train the ANN on the mixtures of experimental data only (ANN_E_ neural networks), without using the simulated spectra. However, in this case, the uncertainty of the results increases strongly (*vide infra*). The details of the ANN architecture, training, and validation are given in Note S2.

Once the ANN is trained, it can be used to rate the different candidate solutions to the MCR problem that are sampled from the AFS. For our model dataset introduced in Fig. 1, the ANN-yielded scores for spectra obtained from different linear combinations of PCs are compared with the true score values (Euclidean distances between the corresponding spectrum and the nearest true pure species spectrum) in Fig. 4. Here, we show the results obtained with ANN_T+E_. As one can see, the score values yielded by the trained ANNs follow closely the dependency of the Euclidean distance between the spectrum of a mixture and the nearest pure spectrum on transformation coefficients *t*_*ij*_. In particular, just by looking at Fig. 4b one can identify three distinct minima regions in the *ξ*(*t*_*i*2_, *t*_*i*3_) map, corresponding to the three different pure species. To obtain the final solution (the best estimate of the sought spectra of pure species), we need to find the set of spectra, for which the combined score (root mean square of individual scores) *ξ̄* is the lowest. To accomplish this, we can use any global optimization scheme, *e.g.*, a simple simulated annealing^65^ optimization. The details of our algorithm are given in Note S3.

**Fig. 4:**
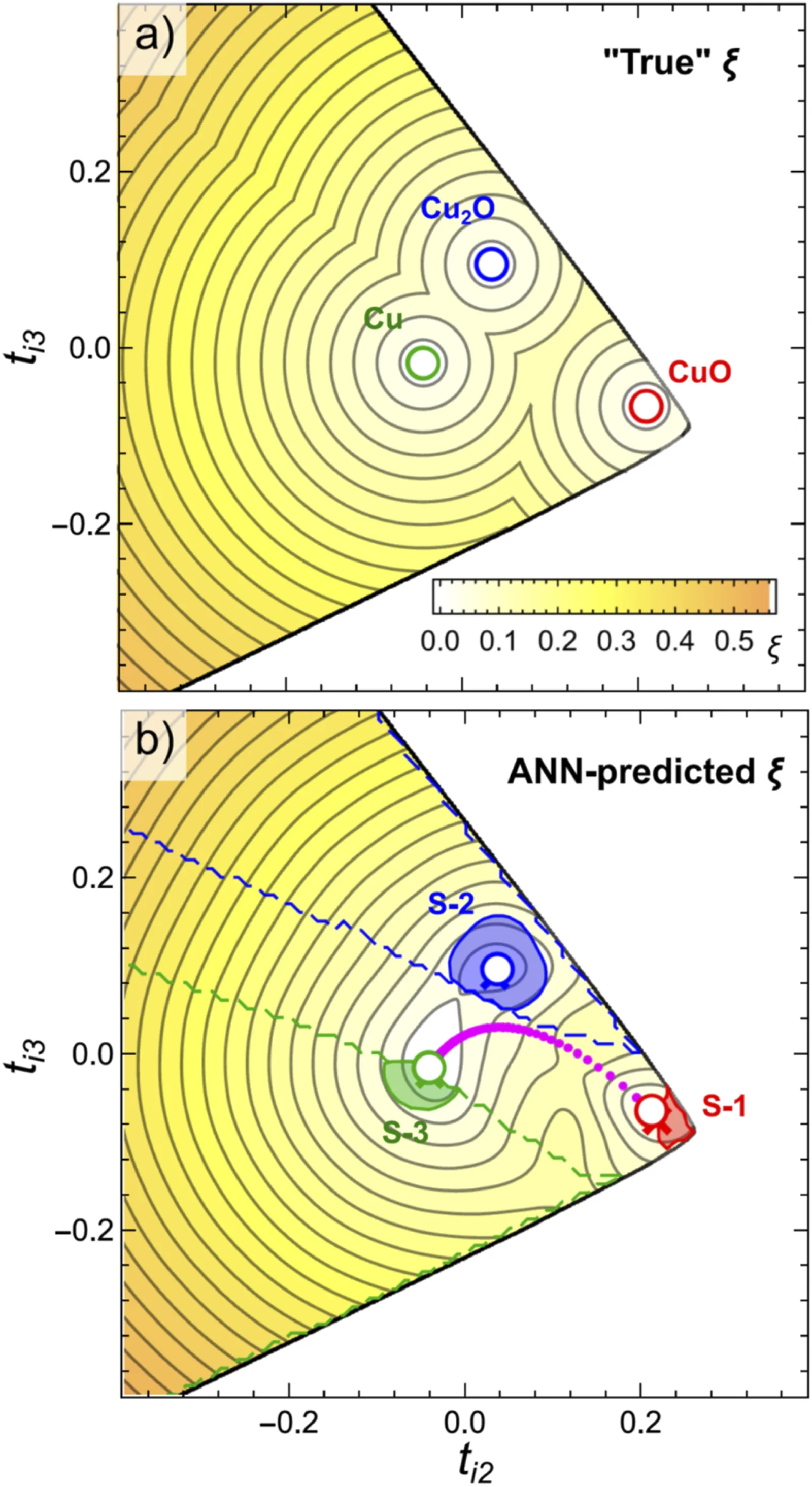
Application of the ANN-MCR approach to model data. (a) Contour plot showing the true dependency of scores *ξ* on the values of the transformation matrix coefficients *t*_*i*2_ and *t*_*i*3_ within the spectra-specific AFS for the dataset shown in Fig. 1a. (b) Corresponding average prediction of 20 ANN_T+E_ neural networks. Green, blue and red crosses in (b) show the average optimal solution to the MCR problem. Green, blue and red regions in (b) show the uncertainty of the obtained results, as obtained by comparing the solutions yielded by 20 separately trained ANN_T+E_ neural networks. Green, blue and red circles in (a and b) show the true solution, corresponding to experimental reference spectra of metallic Cu, Cu_2_O and CuO.

The final ANN-MCR solution for the model dataset introduced in Fig. 1 is shown in Fig. 5. As one can see, our approach reliably recovers the spectra of all pure species (metallic Cu, Cu_2_O and CuO, Fig. 5a), as well as the corresponding concentration profiles (Fig. 5b). It is noteworthy that even the spectrum of Cu_2_O, whose concentration in our dataset never exceeds 50%, is reconstructed very well. Furthermore, we emphasize that the uncertainties of the final solution depicted in Fig. 5 reflect the maximum deviations of different solutions from the average solution provided by the 20 independently trained ANNs. None of these individual solutions deviates too strongly from the average solution, despite the fact that each of the ANNs was exposed to a subset containing different training spectra.

**Fig. 5:**
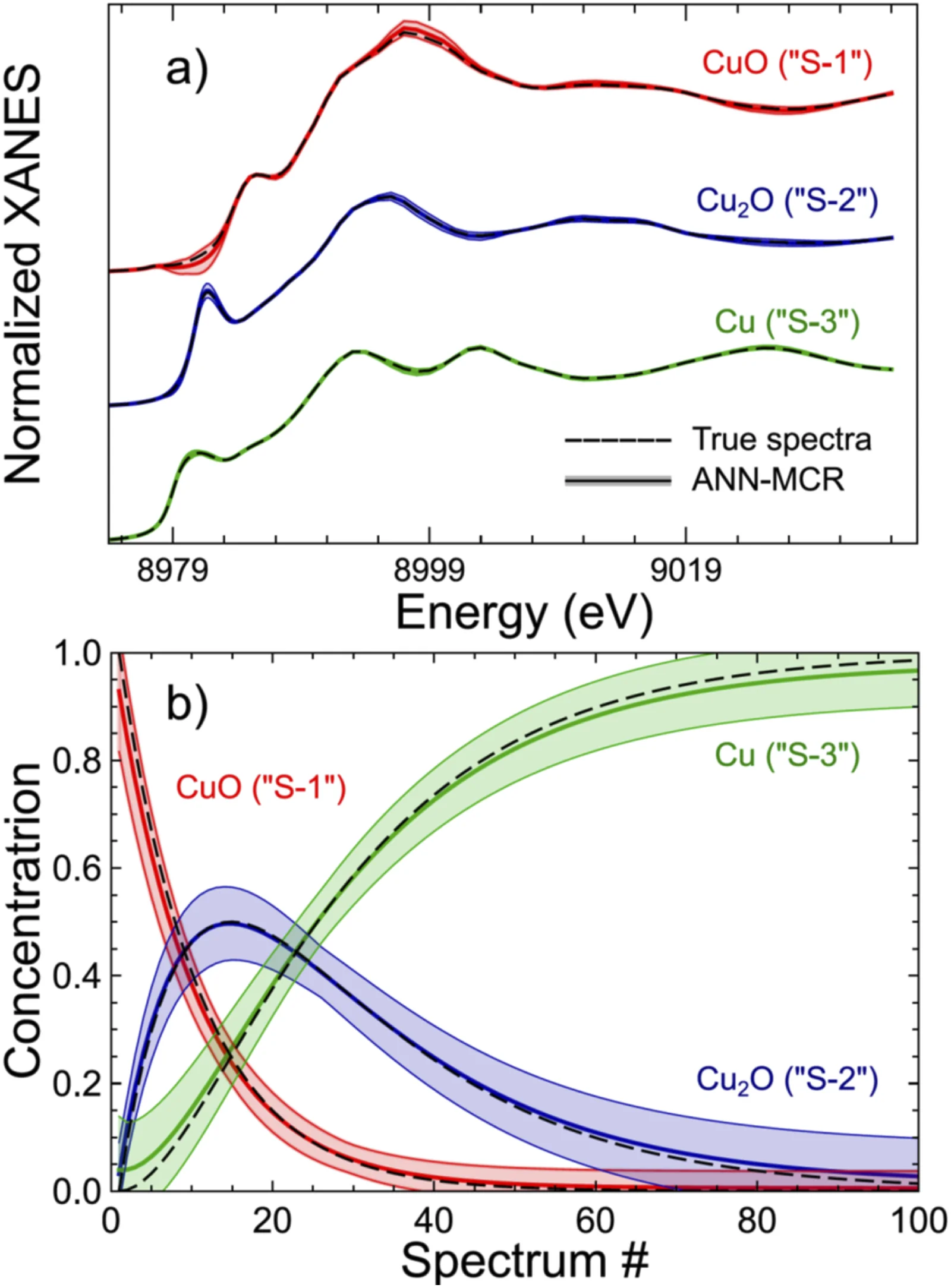
Solution to the MCR problem for model data obtained by the ANN-MCR approach. Spectra of pure species (a) and concentration profiles (b) corresponding to the final (averaged) ANN-MCR solution depicted in Fig. 4 are shown as thick lines. Green, blue and red shaded regions show the uncertainty of the obtained results, as obtained by comparing the solutions yielded by 20 separately trained ANN_T+E_ neural networks. Reference spectra of metallic Cu, Cu_2_O and CuO and the corresponding true concentration profiles are shown with dashed lines.

In particular, while some of the ANNs were exposed to the experimental spectra of metallic Cu, Cu_2_O or CuO during the training, others were not and still provided very similar results. To highlight this, in Fig. S3 we show the ANN-MCR results obtained using ANN_T+E-test_, where none of the ANNs were exposed to the experimental Cu, Cu_2_O or CuO spectra. The obtained result (Fig. S3(a, d and g)) is still very similar to the one in Fig. 5. Fig. S3(b, e and h) show the results obtained using a subset of ANN_T+E_, where all of the trained ANNs were exposed to the experimental Cu, Cu_2_O or CuO spectra during the training (in addition to hundreds of other experimental and FDMNES-generated spectra). Again, the obtained result is very similar to the one in Fig. 5, with only slightly lower uncertainties. The main source of the uncertainty for the result in Fig. S3(b, e and h) is the bias of our ANN models, namely, the insufficient flexibility of our ANNs that prevents them from perfectly memorizing all the training spectra. This uncertainty can be further suppressed, *e.g.*, by increasing the complexity of the model (the size of ANNs). However, this would result in lower ability of our ANNs to generalize, *i.e.*, to robustly evaluate spectra that are not explicitly included in the training dataset. Overall, the comparison of the results from Fig. 5 and S3 strongly suggests that our ANN-MCR works robustly for both the spectra that were used during the ANN training, as well as for the spectra that were not included in the training dataset. This property stems from the fact that the training data were sufficiently diverse and representative for the ANN to learn the overall characteristics of “physically reasonable” Cu K-edge XANES spectra. Clearly, the decrease in the size of the original spectra library from which the training spectra are constructed, would have a negative impact on the ability of our method to generalize. As an illustration of this, in Fig. S3(c, f and g) we show the results obtained by ANN_E_, which were trained only on mixtures of available experimental reference spectra and without including the spectra of metallic Cu, Cu_2_O and CuO (each ANN was still trained on 100 000 mixture spectra in total). In this case, the uncertainties of the obtained solution increase drastically. Thus, one can conclude that the usage of FDMNES-simulated spectra for ANN training, results in more diverse training datasets, which helps significantly to obtain reliable solutions using our ANN-MCR method. We also note that systematic errors in XANES modelling do introduce some uncertainty in the obtained results. However, this is partially compensated by the fact that the final solution is determined not solely by the ANN, but also by the need to satisfy the imposed constraints on spectra and concentrations. Nonetheless, further improvement in the accuracy of the *ab initio* XANES simulations could enhance the accuracy of our approach in the future.

It is also instructive to consider the dependency of the ANN-MCR result accuracy on the concentrations of different species contributing to our dataset. In Fig. S4, we apply our ANN-MCR method to the datasets, constructed analogously to that in Fig. 1, but with different maximum concentrations for the intermediate species (Cu_2_O, see the concentration profiles in Fig. S4(f–j)). The maximal concentrations for Cu_2_O, as yielded by the ANN-MCR method, are compared with the true maximal concentration values in Fig. 6. One can observe that for concentrations of intermediate species of *ca.* 30% and higher, the accuracy and uncertainty of our approach practically does not depend on the concentration profiles. However, for lower concentrations of intermediate species, the uncertainty of the obtained result increases noticeably, and the average solution increasingly deviates from the true solution. The origin of this effect becomes clear by considering the AFS for the situation with the low intermediate species concentration (inset in Fig. 6). Indeed, here the decrease in the spectroscopic contrast between the spectra in the original dataset results in an increased overlap between the AFS for different species. In particular, the solution where the spectrum for S-2 species is very close to the spectrum of S-1 species becomes a viable solution that does not violate the non-negativity constraint for concentration profiles and yields a low score value of *ξ*. Thus, at low concentrations of intermediates, there are two different solutions that both satisfy the imposed constraints and also yield low *ξ̄* score values: (i) the “correct” solution where we have three distinct pure species with the intermediate species having maximal concentration much smaller than the maximal concentrations for two other species, and (ii) the alternative solution where the spectra and maximal concentrations of S-1 and S-2 species become similar. Without additional information, it is impossible to distinguish between these two solutions. We argue that this is true not only for our ANN-MCR approach, but also for any other MCR method. Indeed, both these solutions correspond to plausibly-looking spectra and concentration profiles. This result indicates that extreme caution is needed when applying any MCR methods for the identification of spectra of minority species, even for ideal, noise-free datasets. The ability to identify these situations and signal about them *via* the increased uncertainties of the obtained results is a highly useful feature of our method. Once identified, these ambiguities can be further resolved by imposing additional constraints on concentration profiles or by adding additional auxiliary spectra to the dataset with the concentration of expected intermediate species intentionally increased. We also note that in real cases, in addition to the aforementioned unavoidable uncertainty, the ability of the MCR method to recover the contributions of minority species will be further lowered by the experimental noise and the quality of the spectra alignment. As guidance, for any MCR method to work, the maximum contribution of minority species should be much higher than the contribution of noise or the changes in the spectra due to spectra misalignment.

**Fig. 6:**
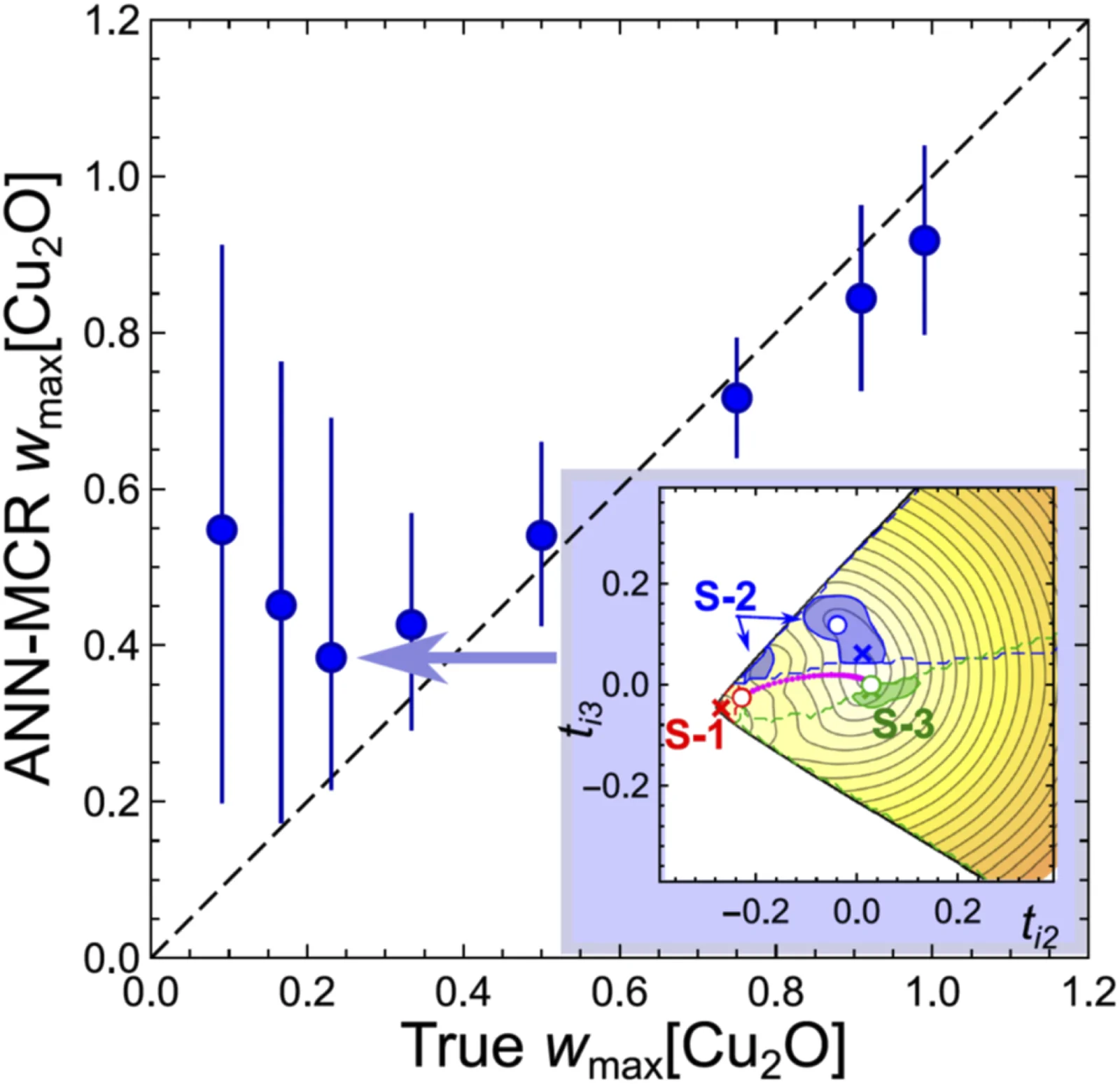
Testing the sensitivity limits of the MCR method. The ANN-MCR method with 20 ANN_T+E_ neural networks was used to recover the spectra of pure species and the corresponding concentration profiles for model mixtures of metallic Cu, Cu_2_O and CuO spectra. Corresponding concentration profiles are shown in Fig. S4f–j. The circles with error bars show the relation between the true maximal concentration of Cu_2_O species and the maximal concentration of Cu_2_O, as obtained by our ANN-MCR method. The inset shows the spectra-specific AFS for the case with true maximal concentration of Cu_2_O set to 25%, with the contour plot showing the dependency of the ANN-yielded score on the values of the transformation matrix coefficients *t*_*i*2_ and *t*_*i*3_. Green, blue and red crosses in the inset show the obtained average optimal solution to the MCR problem, with green, blue and red shaded regions showing the uncertainty of the obtained results. Green, blue and red circles show the true solution, corresponding to experimental reference spectra of metallic Cu, Cu_2_O and CuO.

Finally, we note that while in this section we demonstrated the applicability of our approach on an example dataset corresponding to mixtures of three species, the ANN-MCR method, in principle, can also be applied to mixtures of any number of species. The application of our ANN-MCR approach to model datasets featuring two and four different species is illustrated in Fig. S5 and S6, respectively.

### 3.4 Application of ANN-MCR to experimental *operando* data

With the accuracy of our approach validated on model datasets, we can now apply it for the interpretation of actual *operando* spectroscopic data, obtained in a time-dependent study of the transformations of Cu-based materials. Here we focus on the intriguing transformations of Cu catalysts under electrocatalytic CO_2_ conversion in acidic electrolytes. This reaction is normally carried out in neutral to alkaline media, while the use of acidic electrolytes has been recently suggested as a pathway to improve the carbon efficiency of the process, *e.g.* by lowering the formation of carbonate species.^66,67^ However, a main concern in acidic media is the decreased stability of the employed catalyst, and specifically for Cu, dissolution is expected over a significant pH-potential range based on its Pourbaix diagram.^66,68^ Still, the reports on the *operando* evolution of Cu catalysts under acidic CO_2_RR conditions remain scarce.

In our experiment, we investigated the stability and evolution of Cu_2_O nanocubes (NCs) in an electrolyte consisting of a mixture of 0.1 M oxalic acid (H_2_C_2_O_4_) and 0.1 M KOH (bulk pH = 1.9). The Cu_2_O NC system has been extensively investigated by us previously,^35,69–73^ but so far only in alkaline (in KOH) or neutral (CO_2_-saturated KHCO_3_) media. We also note that the addition of KOH to the acidic electrolyte is needed to provide alkali metal cations, which are known to play a critical role in enabling the CO_2_RR, and, to some extent, suppressing the competing hydrogen evolution.^68,74^

*Operando* time-resolved XANES data collected for the Cu_2_O NCs in air, in acidic electrolyte under open circuit potential (OCP) conditions, at constant applied current density (−120 mA cm^−2^) and at OCP after the reaction, are shown in Fig. 7a. The raw XAS data before normalization are shown in Fig. S7. Reassuringly, in the oxalic acid-containing electrolyte our catalyst did not fully dissolve. After a quick initial loss of the sample during filling the electrochemical cell with electrolyte, the Cu K-edge XAS signal intensity stabilizes at *ca.* 50% of its initial value and practically does not change over the further 1 hour under CO_2_RR conditions (inset in Fig. S7). The initial loss of the catalyst could be partially linked to the imperfections in sample preparation (spray-coating of a relatively thick sample layer on the GDE).

**Fig. 7:**
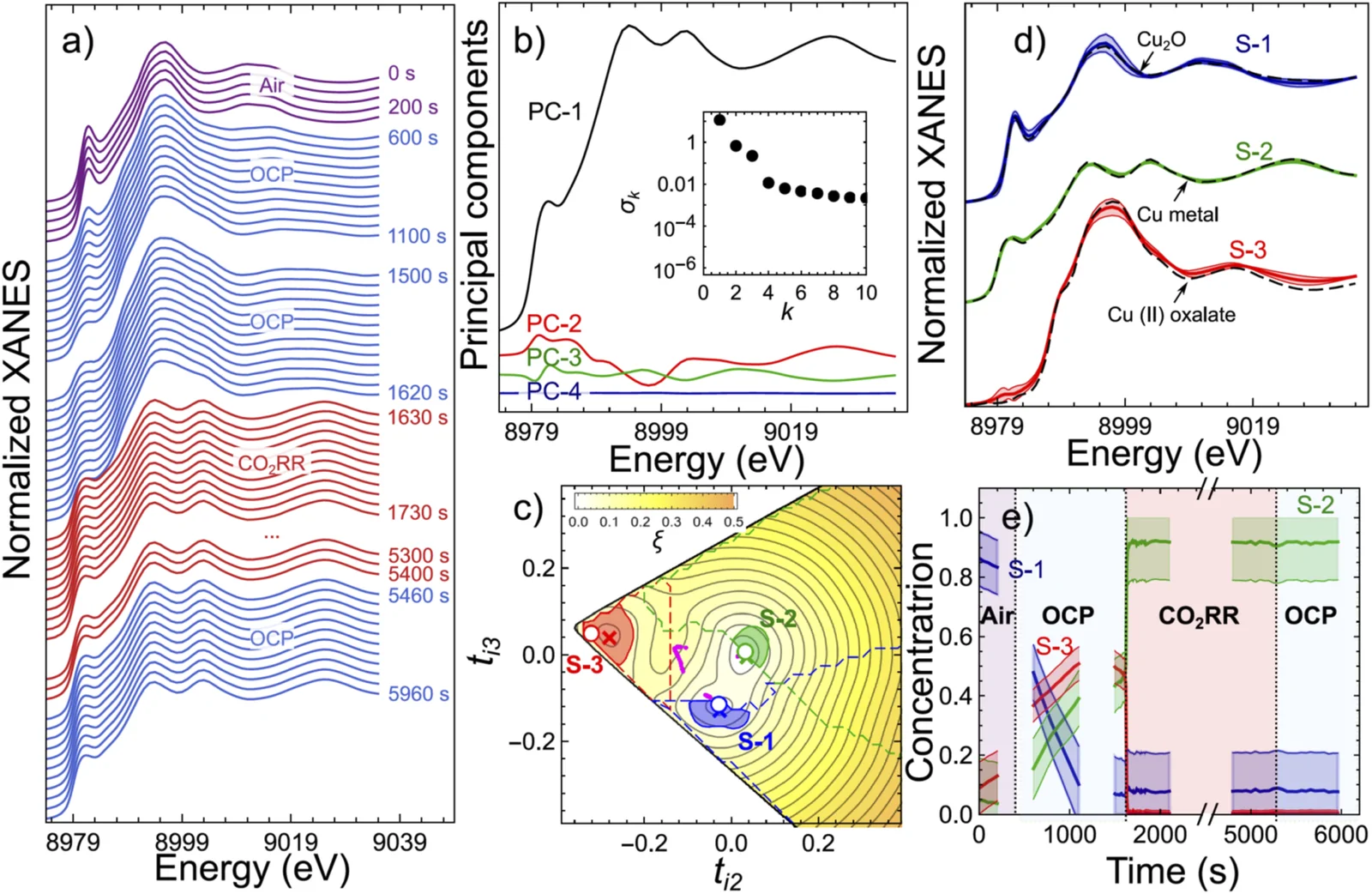
Speciation of Cu_2_O NCs under acidic CO_2_RR conditions. (a) *Operando* time-resolved Cu K-edge XANES spectra collected in fluorescence mode in air, in electrolyte (0.1 M oxalic acid and 0.1 M KOH mixture) at OCP, under CO_2_RR conditions (−120 mA cm^−2^ current density) and at OCP after the CO_2_RR. (b) PCA for the collected dataset: first four PCs, extracted from the dataset in (a), and the “scree-plot” showing the relative importance of different PCs (inset). (c) Spectra-specific AFS for the dataset shown in (a), with the contour plot showing the dependency of the ANN-yielded score *ξ* on the transformation matrix coefficients *t*_*i*2_ and *t*_*i*3_. Red, blue and green crosses show the optimal solution yielding the lowest score. Corresponding pure species spectra and time-dependent concentration profiles are shown in (d) and (e), respectively. Dashed lines in (d) show the reference spectra of Cu_2_O, metallic Cu and Cu(ii) oxalate. The projections of these reference spectra on the extracted PCs are shown with white circles in (c).

The Cu species in our NCs experience significant chemical and structural transformations. While the XANES spectrum of the as-prepared catalyst in air resembles the one for the Cu_2_O reference, in electrolyte rapid changes are observed (Fig. 7a). Intriguingly, these changes take place already under open circuit conditions, without any potential being applied. Under CO_2_RR conditions (−120 mA cm^−2^) the catalyst once again changes and appears to be reduced.

To understand and quantify these changes, we employ our ANN-MCR method. The PCA applied to the obtained dataset (Fig. 7b) strongly suggests that there are three spectroscopically distinct species contributing to the dataset. Indeed, the so-called “scree-plot”, showing the relative importance of different PCs, reveals that the first three PCs explain most of the variations between the individual spectra in the collected dataset and that only the first three PCs contain meaningful spectroscopic features. Based on the extracted PCs, we first construct spectra-specific AFS and then evaluate different solutions to the MCR problem using ANN_T+E_. The dependency of the average ANN-yielded score on the transformation matrix coefficients *t*_*i*2_ and *t*_*i*3_ is shown in Fig. 7c. Again, three distinct minima in this map (marked by red, green and blue crosses in Fig. 7c) can be easily identified and are readily found by the simulated annealing algorithm. Different independently trained ANNs yield slightly different score maps and, hence, slightly different minima points, resulting in the uncertainty of the final solution, as indicated by red, green and blue filled regions in Fig. 7c. The spectra corresponding to the average solution, along with their uncertainties, are shown in Fig. 7d. By comparing the obtained spectra with those in our standard library, the spectra corresponding to species S-1 and S-2 can be readily identified as belonging to Cu_2_O and metallic Cu, as expected. From the position of the absorption edge and the white line intensity, the spectrum for species S-3 can be assigned to some Cu(ii) species. However, it does not match any standard spectra of Cu oxide or hydroxide species. Luckily, a very similar spectrum can be found in the MDR XAS database^41^ and belongs to Cu(ii) oxalate, thus allowing us to assign the S-3 species to the oxalate species formed *in situ*. We highlight here that neither an experimental nor a theoretical spectrum of oxalate was used in the ANN training. Thus the good agreement between the obtained spectrum of S-3 species and that of Cu oxalate is another demonstration of the ability of our method to track appearance of the structures not included in the training dataset.

The concentration profiles extracted by our method from the experimental *operando* data are shown in Fig. 7e. As expected, the as-prepared catalyst is dominated by the contribution of Cu_2_O-like species. However, upon contact with the electrolyte (at OCP), the speciation of the catalyst changes: not only do we observe the formation of oxalate species, but, at the same time, also comparable amounts of metallic Cu are formed. At the end of the measurements under OCP conditions, the contribution of Cu(i) species drops to nearly zero, with half of the remaining catalyst converted to a metallic state and the other half – to Cu(ii) oxalate species. This suggests that Cu(i) species are not stable in oxalic acid containing electrolyte and the transformation of Cu(i) species is a charge disproportionation reaction (2Cu(i) → Cu(0) + Cu(ii)). Surprisingly, there are not many references to such a process in the prior literature. Nonetheless, it has been reported that Cu(i) oxalates can form upon the reaction of Cu_2_O with oxalic acid and that these Cu(i) oxalates can indeed undergo a further disproportionation reaction, forming Cu(ii) oxalates and metallic Cu.^75^ We hypothesize thus that Cu_2_O species at OCP are first converted into Cu(i) oxalates, which are too short-lived to be detected by our method, which then, in turn, quickly form Cu(ii) species and metallic Cu. This process could also be partially responsible for the observed stability of our catalyst in acidic media and, thus, distinguish the effect of oxalic acid from that of electrolytes containing other strong inorganic acids.

Upon applying current, the chemical state and the structure of the catalyst change again, as the oxalate species undergo fast reduction, also forming metallic Cu species. This process occurs within less than 30 seconds, after which the catalyst is fully converted into a metallic state. We note that no further changes in the catalyst are observed during the 1 hour of the CO_2_RR. Furthermore, when returning to OCP, the catalyst still remains fully metallic and does not dissolve (Fig. 6e and S7).

We also note that, in Fig. 7e, some gradual increase in the concentration of Cu(ii) species can be observed in the as-prepared catalyst, when it is measured in air. While our ANN-MCR method assigns this species to oxalate, clearly, no such oxalate formation is expected for the catalyst that is not in contact with electrolyte. Instead, this increase in Cu(ii) species is attributed to beam damage, which we have observed also in our previous studies^76^ and is related to minor oxidation of Cu_2_O NCs when exposed to the X-ray beam in air.^77^ Since the contribution of these Cu(ii) oxide species is always low, our approach, when applied to the entire dataset, does not distinguish them as a new species but rather lumps their contribution with that of oxalate species, which are formed at much higher concentrations during the later stages of our experiment. To discriminate between the contributions of these two different Cu(ii) species, in Fig. S8, we apply our ANN-XANES approach to the subset of our experimental spectra consisting only of the spectra collected in air. Our method, in this case, confirms the coexistence of Cu_2_O-like species with small amounts of Cu(ii) species. The extracted spectrum of these Cu(ii) species features an intense white line, but lacks the characteristic shoulder at *ca.* 8989 eV, which is characteristic of the spectrum of oxalate and could be linked to the presence of square-planar structural motifs.^64^ We thus assign the obtained spectrum to Cu(ii) species in a distorted octahedral environment, whose contribution slightly increase upon prolonged exposure to X-ray beam in air.

Finally, since, in our experiment, in addition to XANES data also full EXAFS spectra were collected, we can use the insight from EXAFS data fitting to further validate the speciation yielded by our ANN-MCR method. The raw EXAFS spectra and their fits in *k*- and *R*-spaces are shown in Fig. S9. The evolution of structural and non-structural fit parameters of the NCs as extracted from EXAFS fitting is shown in Fig. S10.

EXAFS fitting results confirm nicely the conclusions obtained from the ANN-MCR-XANES analysis. EXAFS data for the as-prepared NCs are dominated by a Cu–O contribution with a coordination number of *ca.* 2.0 and a short Cu–O interatomic distance of *ca.* 1.83 Å, which agrees well with the structural parameters for Cu_2_O.^35^ Upon exposure to the electrolyte, the Cu–O coordination number first increases and then decreases upon appearance of a Cu–Cu contribution to the EXAFS data. The Cu–O bond length noticeably increases in this process. At the end of our measurements at OCP (immediately before current is applied), the Cu–O coordination number is 2.2 ± 0.2 and Cu–Cu coordination number is 6.8 ± 0.2, which agrees well with a structural model where half of the Cu species form a metallic structure (with 12 Cu neighbours for each Cu atom in an ideal fcc lattice), and the other half forms a 4-coordinated complex with Cu–O bond lengths of 1.89 ± 0.01 Å. The latter parameters match well with the reported structure for Cu(ii) oxalate.^78^ Under CO_2_RR conditions, the Cu–O contribution rapidly disappears, and the spectra are dominated by a Cu–Cu contribution with a final coordination number of *ca.* 11 ± 0.1. Upon switching back to OCP conditions, no changes in the EXAFS parameters are observed.

EXAFS and ANN-MCR results can be compared quantitatively. Indeed, if *w*_Cu_, *w*_Cu_2_O_ and *w*_Oxalate_ are concentrations for metallic Cu, Cu(i) and Cu(ii) species, respectively, then the expected average Cu–Cu and Cu–O coordination numbers are CN_Cu–Cu_ = *N*_Cu_*w*_Cu_ and CN_Cu–O_ = *N*_Cu_2_O_*w*_Cu_2_O_ + *N*_Oxalate_*w*_Oxalate_, respectively. Here *N*_Cu_ is the number of atoms in the metallic fcc lattice (12 in an ideal lattice), *N*_Cu_2_O_ is the number of nearest oxygen neighbours for Cu in the Cu_2_O structure (2) and *N*_Oxalate_ is the number of nearest neighbours in the oxalate structure (4). As shown in Fig. 8, CNs extracted from EXAFS analysis and those estimated from the XANES data are in excellent agreement. Furthermore, the average Cu–O interatomic distances extracted from EXAFS data and estimated from ANN-MCR results using an analogous approach, also match well. Thus, EXAFS analysis fully confirms the results that we have obtained by the ANN-MCR method and validates the accuracy of our approach.

**Fig. 8:**
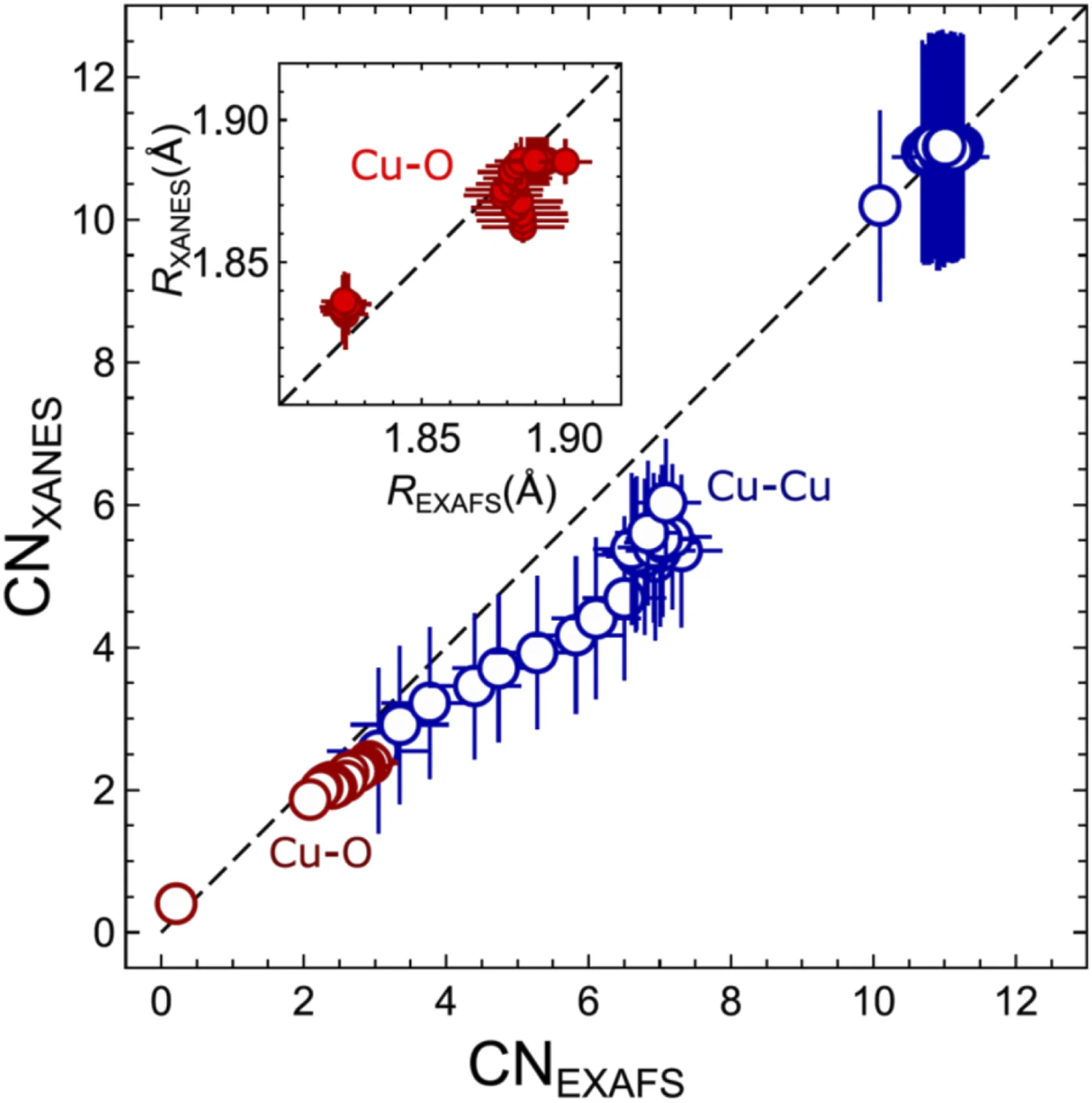
Validation of ANN-MCR results with EXAFS fitting. Average Cu–O (red circles) and Cu–Cu coordination numbers (blue circles), as extracted from EXAFS fits, are compared with average coordination numbers, as estimated from ANN-MCR-derived concentration profiles of metallic Cu, Cu_2_O and oxalate species. The inset shows the average Cu–O interatomic distance, as extracted from EXAFS fits, with the average Cu–O distance as estimated from ANN-MCR-derived concentration profiles of Cu_2_O and oxalate species.

## 4. Conclusions

In this work, we demonstrated that a data-driven approach can be successfully used to facilitate the disentanglement of distinct spectroscopic contributions to experimental datasets, arising from the *operando* measurement of heterogeneous samples. Through the example of the interpretation of Cu K-edge XANES data, we have shown that pure spectra for a broad range of different compounds share similarities that can be readily recognized by supervised machine learning methods. This narrows dramatically the space of possible solutions to the spectral decomposition problem. At the same time, our approach allows us to easily quantify the uncertainties of the obtained result, which is a crucial, but often overlooked, aspect in the existing applications of MCR methods. In particular, we have demonstrated that the application of MCR methods for the identification of minority species should be done with caution and cannot be fully automatized, since multiple equivalently plausible solutions arise in these scenarios.

However, in situations where transient species contribute to the dataset in substantial amounts, our approach provides an easy-to-use pathway for the interpretation of spectroscopic datasets, recovering reliably both the concentrations and the spectra of pure species. The latter can be then assigned to specific structural motifs either through existing databases, *via* EXAFS data analysis, or by further *ab initio* simulations and XANES fitting.^27^ As an example, we have demonstrated that our approach can be very powerful for identifying new chemical processes, such as reduction *via* charge disproportionation, observed here for a Cu_2_O-derived CO_2_RR electrocatalyst in an oxalic acid-containing electrolyte. We emphasize, however, that in more complicated cases, the identification of the specific structural motifs corresponding to the extracted spectra itself can be a highly non-trivial task that requires complementary experiments and theoretical modelling.^30,58^ Nonetheless, the ability to reliably recover the spectra of pure species, provided by our approach, serves as an important first step toward the speciation of heterogeneous materials. This is especially useful for tracking the chemical transformation of catalytic materials under reaction conditions. Nonetheless, the applicability of our approach is not limited to problems in catalysis science and it can be similarly used for the interpretation of time- and spatially-resolved data in a broad range of heterogeneous materials.

## Author contributions

J. Timoshenko and B. Roldan Cuenya co-wrote the paper. J. Timoshenko performed data analysis and developed the data analysis methodology, with input from A. Martini. J. Timoshenko and M. Monteiro designed *operando* experiments. L. De Angelis performed sample synthesis and preliminary characterization. J. Timoshenko, A. Martini, M. Rüscher, L. Bai, L. De Angelis, and M. Monteiro performed synchrotron-based *operando* experiments.

## Conflicts of interest

There are no conflicts to declare.

## Supplementary Material

SC-017-D6SC03428D-s001

SC-017-D6SC03428D-s002

## Data Availability

The data supporting this article have been included as part of the supplementary information (SI). Supplementary information: details of spectra alignment, neural network training, and employed optimization algorithms, as well as supplementary figures. See DOI: https://doi.org/10.1039/d6sc03428d.
