# Supplementary material for: Guiding blind signal separation in X-ray absorption spectroscopy using supervised machine learning
Source: Chem Sci. 2026 Jul 23;17(37):17738–52. doi: 10.1039/d6sc03428d (PMC13435674; doi:10.1039/d6sc03428d)
Supplement: Supplementary file 1 [file SC-017-D6SC03428D-s002.pdf]

Supplementary Information (SI) for Chemical Science.
This journal is © The Royal Society of Chemistry 2026

Guiding Blind Signal Separation in X-ray Absorption Spectroscopy by Supervised Machine Learning

Janis Timoshenko,[1,*] Andrea Martini,[1] Martina Rüscher,[1] Lichen Bai,[1] Leonardo Domenico De Angelis,[1,2] Mariana Cecilio de Oliveira Monteiro[1] and Beatriz Roldan Cuenya[1,*]

[1]Department of Interface Science, Fritz-Haber Institute of the Max-Planck Society, 14195 Berlin, Germany.

[2]Instituto de Química, Universidade de São Paulo, Av. Prof. Lineu Prestes 748, 05508-900, São Paulo, SP, Brazil.

*Emails: janis@fhi-berlin.mpg.de , roldan@fhi-berlin.mpg.de

Supplementary Information

Supplementary Note 1: Semi-supervised alignment of FDMNES-calculated spectra

The fact that the simulated XANES spectra are usually shifted by an unknown energy shift with respect to the experimental data is one of the main limitations in using ab initio simulations for the interpretation of experimental datasets. This applies not only to the XANES simulations by the FDMNES code,[1, 2] but also to other popular codes such as, e.g., FEFF[3-5]. Importantly, the energy shift required to align the simulated spectra with experimental data depends on the material. As a result, generally one cannot use the same energy shift to align the spectra calculated for different materials. For example, as shown in **Figure S1**, in the case of our FDMNES simulations, the required energy shift ΔE_1 for different materials is typically within the range between -4 and +4 eV. The procedure for the spectra alignment is discussed below. Note that ΔE_1 values were calculated after the FDMNES-specific pre-alignment step, where the energy grids for all calculated spectra were first corrected by the corresponding EPSII value (the reference energy of the core orbital, calculated and reported by the FDMNES code).[6]

While ΔE_1 varies between different materials, it is also not a random number. Indeed, as is clear from **Figure S1**, FDMNES-simulated spectra for similar materials need to be shifted by similar value. For example, for most compounds where Cu is in the 2+ oxidation state FDMNES-simulated spectra need to be shifted by ca. +1 or +2 eV, while for most of the Cu(I) materials the required energy shift is negative and can be as low as -4 eV. Assuming that the XANES spectrum itself can be considered as a convenient descriptor of the structural and electronic properties of the material, one can envision that it should be possible to train a supervised machine learning (SML) model that would take the original simulated spectrum as an input and would suggest the necessary energy shift. However, to directly apply such an SML approach, one would need a very large number of training spectra for which the energy shift is known. In our case only for 24 simulated spectra we had the corresponding experimental spectra available, and only for these spectra the energy shift was thus known. To simplify the problem so that the relationship between the spectra and the energy shift can be established based on a small number of labelled training examples, we first perform a dimensionality reduction on the entire set of the simulated spectra, to be able to describe the main differences between the spectra using just one number. The relationship between this descriptor and the necessary energy shift is then established in the next step.

Specifically, the alignment procedure for simulated spectra implemented in our work is as follows:

1) Pre-alignment step: all calculated 3679 Cu K-edge XANES spectra are shifted by $\Delta E_{\mathrm{EPSSII}}$, where $\Delta E_{\mathrm{EPSSII}}$ is the difference between EPSII parameters in the FDMNS calculations for given material and for bulk Cu metal (calculated using the same FDMNES version).
2) All calculated spectra are re-interpolated on the same energy mesh spanning the range between $E_{\mathrm{min}} = E_0$-7 eV and $E_{\mathrm{max}} = E_0$+56 eV, where E_0 = 8979 eV is the tabulated value for the onset of Cu K-edge in metallic Cu. Spectra that are too short after this step are removed. We end up with 3167 spectra that can be used further.
3) The reinterpolated spectra are renormalized by dividing them by their Euclidean norms $\left(\sum_k \mu_i^2(E_k)\right)^{0.5}$ (see the discussion in the main text).
4) 24 FDMNES spectra, for which corresponding experimental data are available, are shifted by additional ΔE_1 (different for different materials), to align the positions of the absorption edges in experiment and simulation. Before alignment, the experimental spectra are reinterpolated and renormalized in the same way as theoretically simulated spectra. We note that despite the overall good agreement between the simulated and experimental data, a perfect match between simulations and experiment is not expected. These systematic errors will affect the data alignment, and different employed algorithms can result in different ΔE_1 values. For example, one can choose ΔE_1 as the difference between

maxima of the first derivatives for simulated and experimental data, or as the difference between energies, where the normalized absorption in experiment and simulation reaches certain value, or as the value that minimizes the Euclidean distance between the experimental and shifted theoretical spectra, etc. Dependently on the further application of the simulated spectra, one or another approach could be more beneficial. In our case the goal is to use the simulated spectra for the interpretation of the principal components extracted from experimental datasets. Therefore, we choose ΔE_1 values as such that minimizes the difference between the projections of experimental and theoretical spectra, when projected on the principal components extracted from experimental data. Specifically, $\Delta E_{1,i}$ for a theoretical spectrum $\mu_{FDMNES,i}(E)$ and corresponding experimental spectrum $\mu_{exp,i}(E)$ is found as the value that minimizes $\xi_i = \sum_j \left(\mu_{FDMNES,i}(E + \Delta E_{1,i}) \cdot v_{ij2}(E) - \mu_{exp,i}(E) \cdot v_{ij2}(E)\right)^2$. Here "·" denotes inner product, and $v_{ij2}(E)$ is the 2nd principal component, obtained by performing the singular value decomposition of a matrix with two rows, where the first row is the $\mu_{exp,i}(E)$ spectrum, and the second row is set to some other experimental spectrum $\mu_{exp,j}(E)$ in our library (in the sum above we sum over all possible choices for this second spectrum $\mu_{exp,j}(E)$). We use here the 2nd principal component only, since the projections of normalized XANES spectra on the first principal component are all nearly equal (see the discussion in the main text).

5) We perform the dimensionality reduction for the full dataset containing 3167 theoretically calculated spectra. Again, different algorithms can be used here. We found that good results can be obtained by using even the simplest approach based on principal component analysis. Namely, we perform singular value decomposition of the matrix containing all 3167 spectra (after the reinterpolation and renormalization), to obtain a set of principal components $v_i(E)$. We then project all the spectra on the 2nd principal component, to obtain a scalar value $a_i = \mu_{FDMNES,i}(E) \cdot v_2(E)$. The obtained a_i values are summarized in **Figure S1**, and are all in the range between ca. -0.3 and +0.3. They can be clearly linked to the local structure and chemical state of the Cu species in the material. In particular, most negative a_i values correspond to Cu(0) compounds, while Cu(II) compounds tend to have positive a_i values. Cu(I) compounds, in turn, tend to have a_i values that are close to 0. Importantly, the fact that the a_i values obtained for our 3679 materials are almost evenly distributed within the aforementioned range suggest that this dataset represents well all the possible spectra for Cu species.

6) As one can see from **Figure S1**, from the results obtained for our 24 spectra for which we have corresponding experimental data available, there is a clear relationship between the a_i values and the corresponding necessary energy shift $\Delta E_{1,i}$. We interpolate this relationship by fitting the ($\Delta E_{1,i}$, a_i) points with a polynomial, defined in the range between a_{min} = -0.11, and a_{max} = 0.19. The former value corresponds to the a_i value for metallic Cu alloys, such as CuNi, and is the lowest from the 24 a_i values for the spectra that have corresponding experimental data available. Similarly, the a_{max} value, (corresponding to CuF_2) is the largest from these 24 a_i values. Here for the interpolation we used a polynomial of 6th degree, as determined by cross-validation procedure by repeatedly randomly splitting the set of labelled data into a training set (21 spectra) and a validation set (3 spectra). If the chosen polynomial degree is too low, the model will not be able to capture the relationship between the spectra and energy shifts even for the training set. Increasing the polynomial degree clearly improves the predictions for the training set, but after a certain point (when overfitting onsets), the performance becomes much worse for the validation set. In our case, the chosen 6th polynomial degree provides a reasonable compromise that provides good performance of our method both, for the training and validation datasets. The final error of the alignment depends strongly on the material. The largest deviation between the energy shift predicted by our semi-supervised model and the actual observed shift between experiment and simulations in our training data is about 1.9 eV. However, such large discrepancies are observed only for some specific material classes (e.g., halogenides such as CuCl and $CuBr_2$). On average, the aforementioned deviation is much lower, namely ca. 0.7 eV for cationic Cu species, and only 0.1 eV for compounds with metallic copper. Thus, while for the datasets featuring halogenides our approach should be applied with caution, for other cases the proposed spectra alignment method is more accurate and appears to be working well. Note that the lower accuracy of the alignment achieved by our method for certain materials stems from the aforementioned fact that in our semi-supervised approach we reduce the dimensionality of the problem to just one dimension, namely, each spectrum is characterized only by a single parameter "a". Clearly, increasing the dimensionality of our latent space (e.g., assigning 2 or 3 numbers to characterize each spectrum) would enable better discrimination between different spectra, and would improve the performance of our alignment procedure. Unfortunately, such an increase in the dimensionality of the problem would also require an exponentially larger number of labelled training examples to fit reliably the interpolation function. In this case, having just 24 spectra with known true energy shifts would not be sufficient anymore, and a much larger number of reliable experimental spectra for materials with well-defined known structures would be necessary.

7) All the spectra in the original data are then shifted by the $\Delta E_{1,I}$ value, as predicted by our model constructed in step (6) based on the corresponding a_i value. We keep only the spectra, for which the a_i values are in-between a_{min} and a_{max}, to avoid errors due to the extrapolation of our model to the regions for which we have no experimental data available. We also remove from the further consideration the spectra that after the alignment are too short, namely, where the maximal E value is lower than E_{max}, or where the minimal E value is higher than E_{min}. All the spectra are, again, reinterpolated and renormalized in this energy range. At the end of this procedure, we end up with 1994 theoretically calculated, reinterpolated and renormalized spectra which can be used to guide the MCR process.

Supplementary Note 2: Training and validation of artificial neural network

To establish the relationship between a spectrum for a mixture and the corresponding score value ξ, we use a training dataset consisting of 100 000 training spectra, obtained from randomly selected pairs of FDMNES-generated spectra, as described in the main text. Using this training set, we train an artificial neural network (ANN). Construction and training of ANN is performed using *Wolfram Mathematica* version 14.0.[7] Our ANN consists of an input layer with 117 nodes (corresponding to 117 energy points between E_{min} and E_{max} in our discretized XANES spectra), an output layer with a single node (yielding the corresponding value of ξ), and three hidden layers with 300 nodes each. To reduce overfitting of the training spectra, we rely on a self-normalizing neural network approach,[8] by using scaled exponential linear units (SELU) as activation functions, and applying 1% alpha-dropout. Training is performed for 300 rounds, with batch size 2048. The loss function is defined as L_2-norm between ANN output and the true score value ξ, averaged over all training examples in the batch. For optimization of ANN weights, the "ADAM" method (stochastic gradient descent with adaptive learning rate) is used with default parameters β_1=0.9 and β_2 = 0.999. Optimal values for hyperparameters (e.g., number of ANN layers and nodes in each layer, dropout percent, batch size, number of training rounds) are obtained by a manual grid search, with the goal to optimize the performance of the ANN on a set of spectra that were not used for ANN training (validation set, constructed analogously to the training set). Overall, the performance of our ANN is not too sensitive to the values of these hyperparameters, and good results can be achieved with quite different architectures. Training is performed on GPU (NVIDIA RTX 4000 Ada). To train a single ANN, ca. 25 min are required.

To estimate the uncertainties of the NN-yielded RDFs, we train independently several NNs using different subsets of training sets, and compare their predictions. The average value is reported as the final NN-yielded answer, while the maximal deviation of NNs predictions from the average value is used to calculate the error bars. The validation of the accuracy of the predicted score values ξ for mixtures of different number of species is shown in **Figure S2**.

Supplementary Note 3: Numerical optimization using simulated annealing

In our ANN-MCR approach, the best solution to the MCR problem is obtained by minimizing the overall score $\bar{\xi}$, which, in turn, is calculated by evaluating via the trained ANN the candidate spectra for pure species. For the minimization process we rely on simulated annealing approach.[9] Specifically, for a mixture of *n* species, we start with a set of *n* points in *n*-1-dimensional space spectra within the AFS that correspond to the *n* candidate spectra and that satisfy the imposed constraints. We then calculate the corresponding $\bar{\xi}$ value as $\bar{\xi}_{old} = \left(\sum_i \xi_i^2\right)^{0.5}$ An update of points coordinates is then proposed, where all *n* points are slightly and randomly moved, and the $\bar{\xi}$ value is recalculated, to obtain the $\bar{\xi}_{new}$ value. If the applied point move violates the non-negativity constraints for spectra or concentrations (or any other constraints the user wants to enforce), the $\bar{\xi}_{new}$ value is penalized by adding a very large number. If $\bar{\xi}_{new}$ values is lower than $\bar{\xi}_{old}$, the proposed move is accepted. If $\bar{\xi}_{new}$ is higher than $\bar{\xi}_{old}$, the proposed move can still be accepted with some probability $p(t) = \exp\left((\bar{\xi}_{old} - \bar{\xi}_{new})/\tau(t)\right)$, to escape local minima and find globally optimal solution. The process is repeated many times (in our case, we perform t_{max} = 10 000 iterations for mixtures of 3 species, and 100 000 iterations when deciphering mixtures of 4 species). The scaling parameter τ is exponentially decreased during the optimization run, so that during the first iterations almost any move that satisfies the constraints is accepted, while at the end of the run only the moves that improve the $\bar{\xi}$ value are accepted. By decreasing the τ value sufficiently slowly, the search algorithm converges to the globally optimal solution.[9] In our case, we decrease τ according to the following "cooling schedule": $\tau(t) = 0.01\exp(-10\ t/t_{max})$.

Supplementary Figures

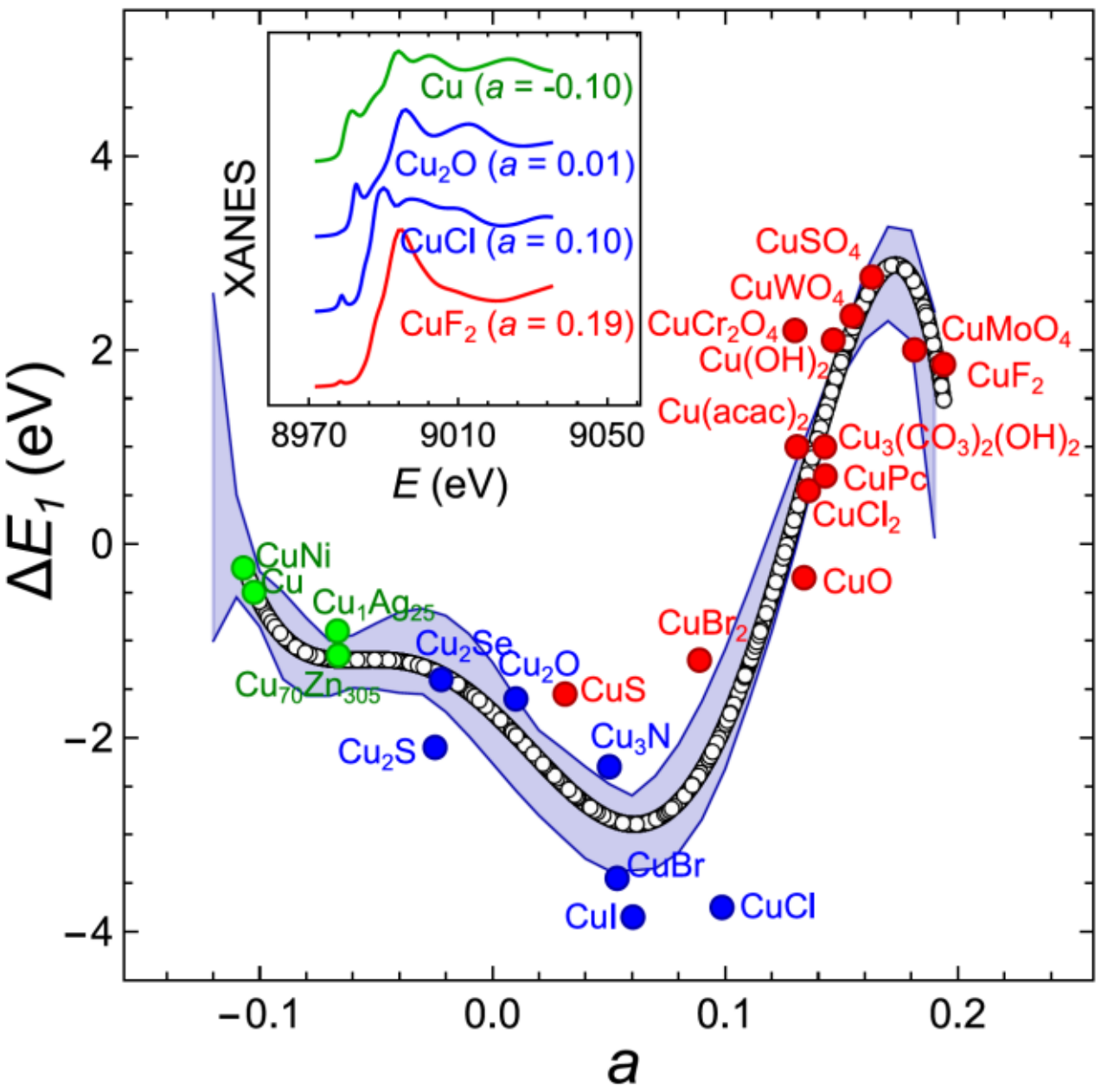

Figure S1. Semi-supervised alignment of simulated spectra. Necessary energy shifts ΔE_1 as obtained by comparing 24 experimental Cu K-edge XANES spectra with corresponding simulation results, and their relation to the projection *a* of the corresponding theoretically simulated spectra on the 2nd PC, extracted from the entire dataset consisting of 3679 calculated Cu K-edge XANES spectra for different compounds. Results for Cu(0), Cu(I) and Cu(II) compounds are shown with green, blue and red colours respectively. Representative spectra corresponding to different *a* values are shown in the inset. These points were used to construct 6-th order polynomial that interpolates the relationship between *a* and ΔE_1. The *a* values for the all calculated XANES spectra, and the corresponding predicted ΔE_1 values are shown with black circles. The uncertainties of the ΔE_1 prediction are shown with blue shaded region, and are obtained by repeating the construction of interpolating polynomial several times using only 21 instead of 24 spectra, every time leaving out one randomly selected spectrum for Cu(0), Cu(I) and Cu(II) species.

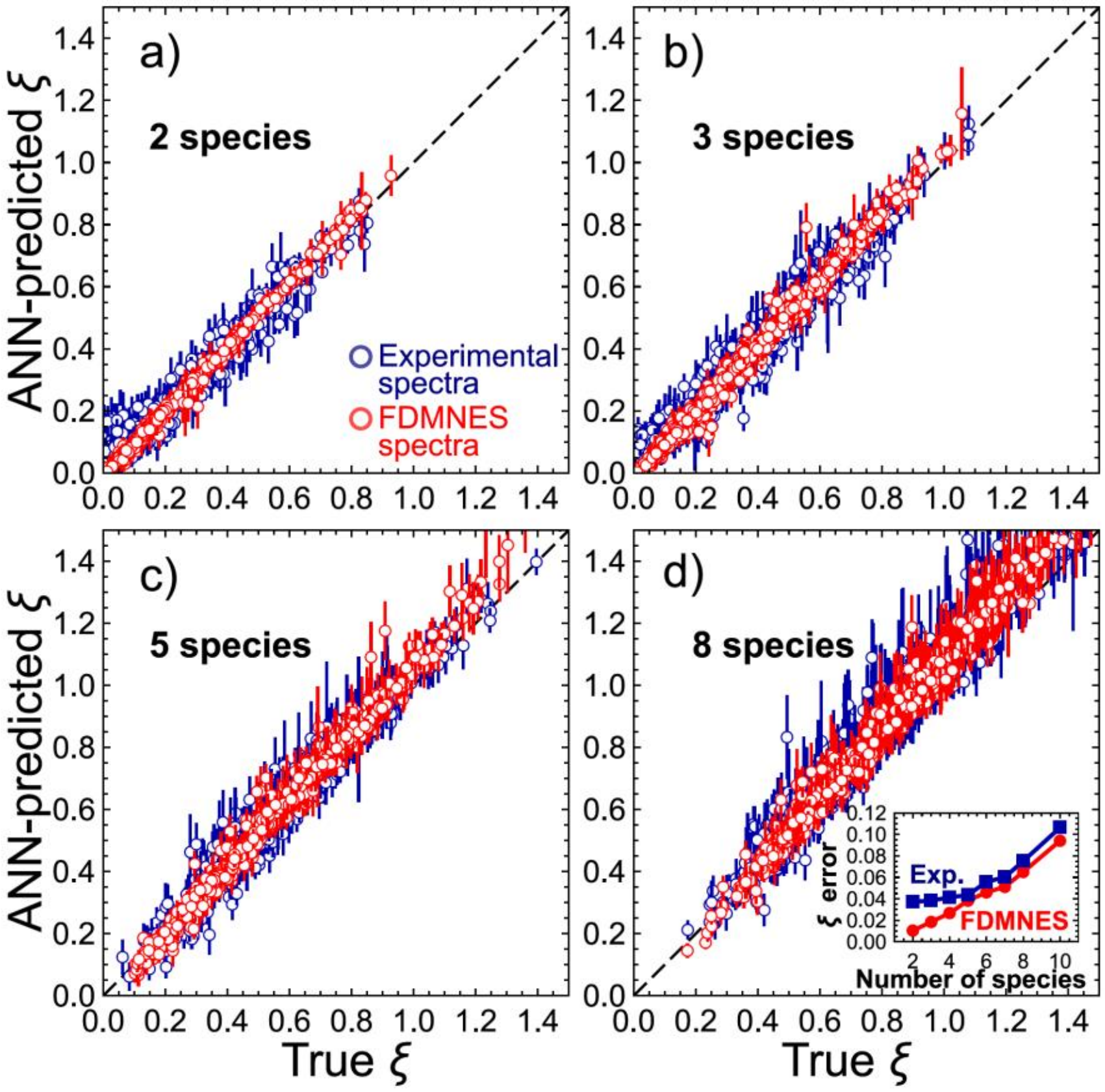

Figure S2. Validation of the accuracy for the trained ANN. The true score values ξ for randomly generated mixtures of 2 (a), 3 (b) 5 (c) or 8 species (d) are compared with the predictions of ANN_T neural network. ANN_T neural networks are trained on mixtures of theoretical data obtained in FDMNES simulations, but can be equally well used to evaluate the mixtures of theoretical spectra (blue circles) and experimental spectra (red circles). Uncertainties reflect the maximal deviations of the predictions of 20 ANN_T neural networks, each trained on a different subset of theoretical data. Only pairwise mixtures of theoretical spectra are used for training, However, the ANNs show reasonable performance in evaluating mixtures of at least up to 8 different species. The dependency of the mean absolute error in ξ prediction as a function of number of mixed species in the mixtures of theoretical and experimental spectra is shown in the inset in (d).

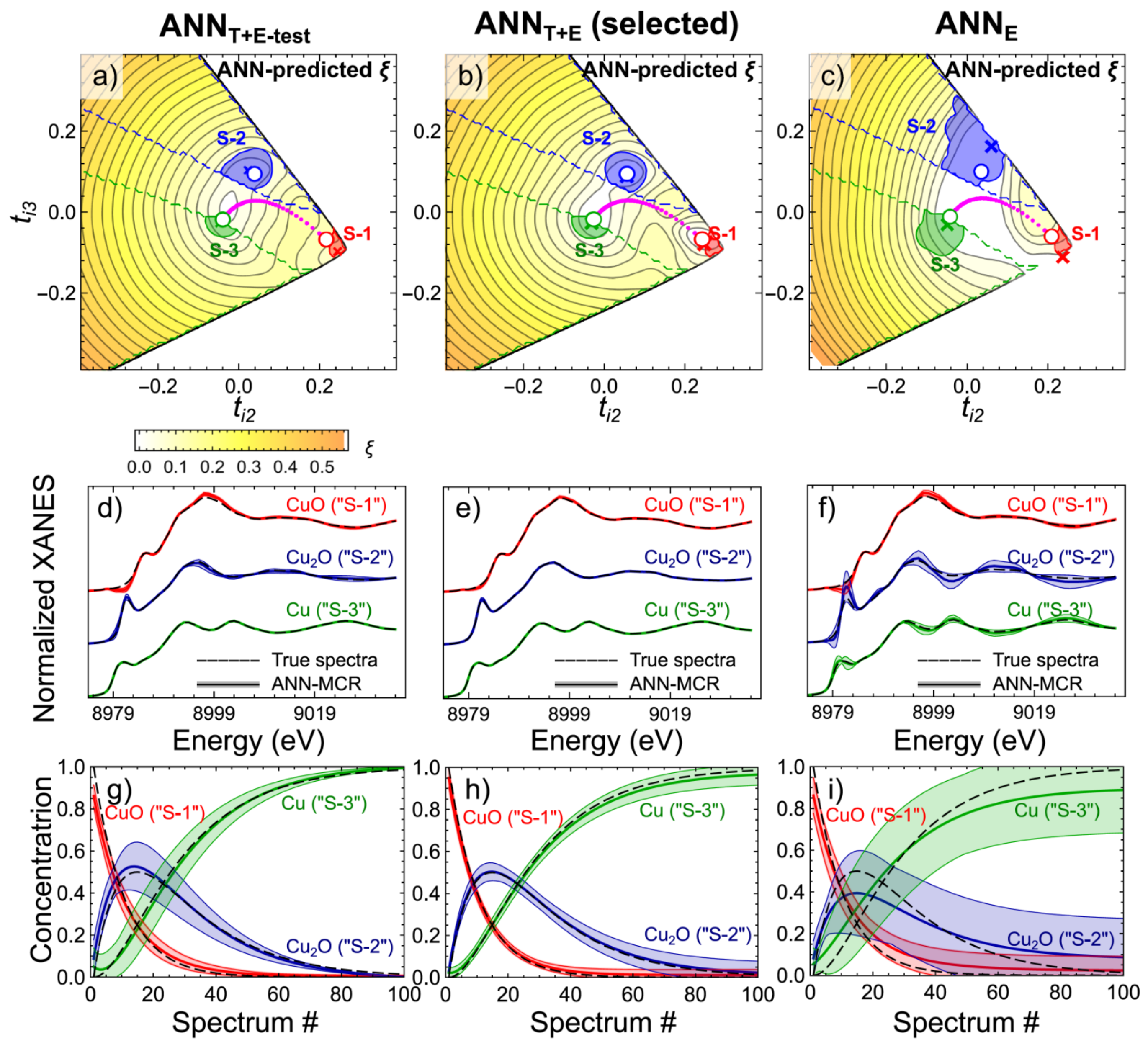

Figure S3. Dependency of the ANN-MCR uncertainties on training data . (a-c) Dependency of scores ξ on the values of t_{i2} and t_{i3} within the spectra-specific AFS for the dataset shown in Figure 1a (a), as obtained from prediction of several ANNs, with corresponding obtained spectra for pure species (d-f) and concentration profiles (g-i). Green, blue and red crosses in (a-c) show the average optimal solution to the MCR problem, with corresponding spectra and concentration profiles shown as thick lines in (d-i). Green, blue and red regions in (a-c), and shaded regions in (d-i) show the uncertainty of the obtained results, as obtained by comparing the solutions yielded by 20 separately trained neural networks. Green, blue and red circles in (a-c) show the true solution, corresponding to experimental reference spectra for metallic Cu, Cu_2O and CuO, with corresponding true spectra and concentration profiles shown in (d-f) and (g-i), respectively. In (a,d,g) we show results obtained with 20 ANN$_{T+E\text{-test}}$, which are trained on simulated and experimental reference spectra, excluding the experimental spectra for Cu_2O, CuO, and Cu metal. In (b,e,h) we show results obtained with 5 selected ANN$_{T+E}$ neural networks, where all of them were exposed to the experimental spectra for Cu_2O, CuO and Cu metal during the training. In (c,f,i) we show results obtained with 20 ANN$_E$ neural networks that are trained on experimental reference spectra excluding the spectra for Cu_2O, CuO, and Cu metal.

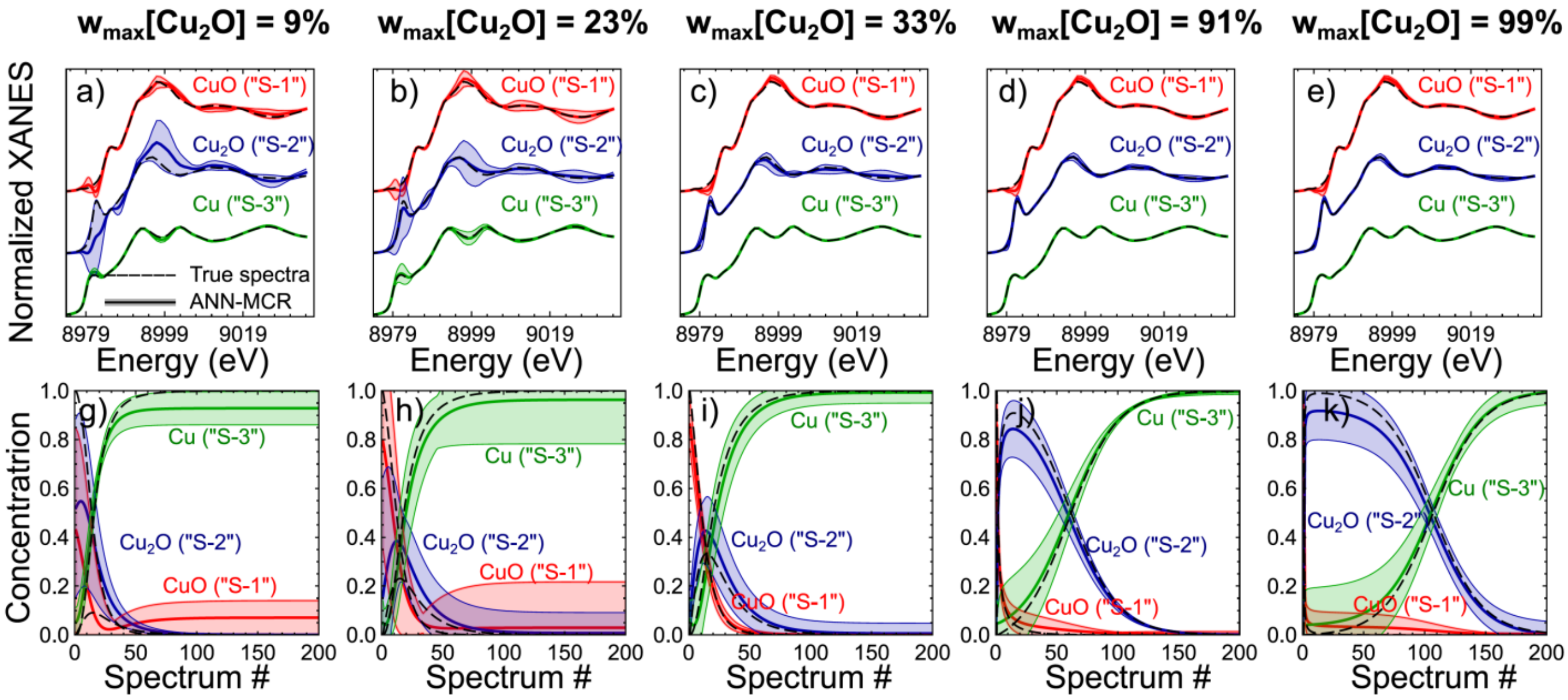

Figure S4. Dependency of the ANN-MCR uncertainties on the maximal concentration for intermediate species. True pure spectra (dashed black lines in a-e) and true concentration profiles (dashed black lines in f-j) are compared with the result obtained by ANN-MCR method. The maximal true concentration of the intermediate Cu_2O species increases from 9% in (a,f), to 99% in (e,j). The maximal concentrations of metallic Cu and CuO in all cases in 100% (complete conversion). Green, blue and red shaded regions show the uncertainty of the obtained results, as obtained by comparing the solutions yielded by 20 separately trained ANN_{T+E} neural networks.

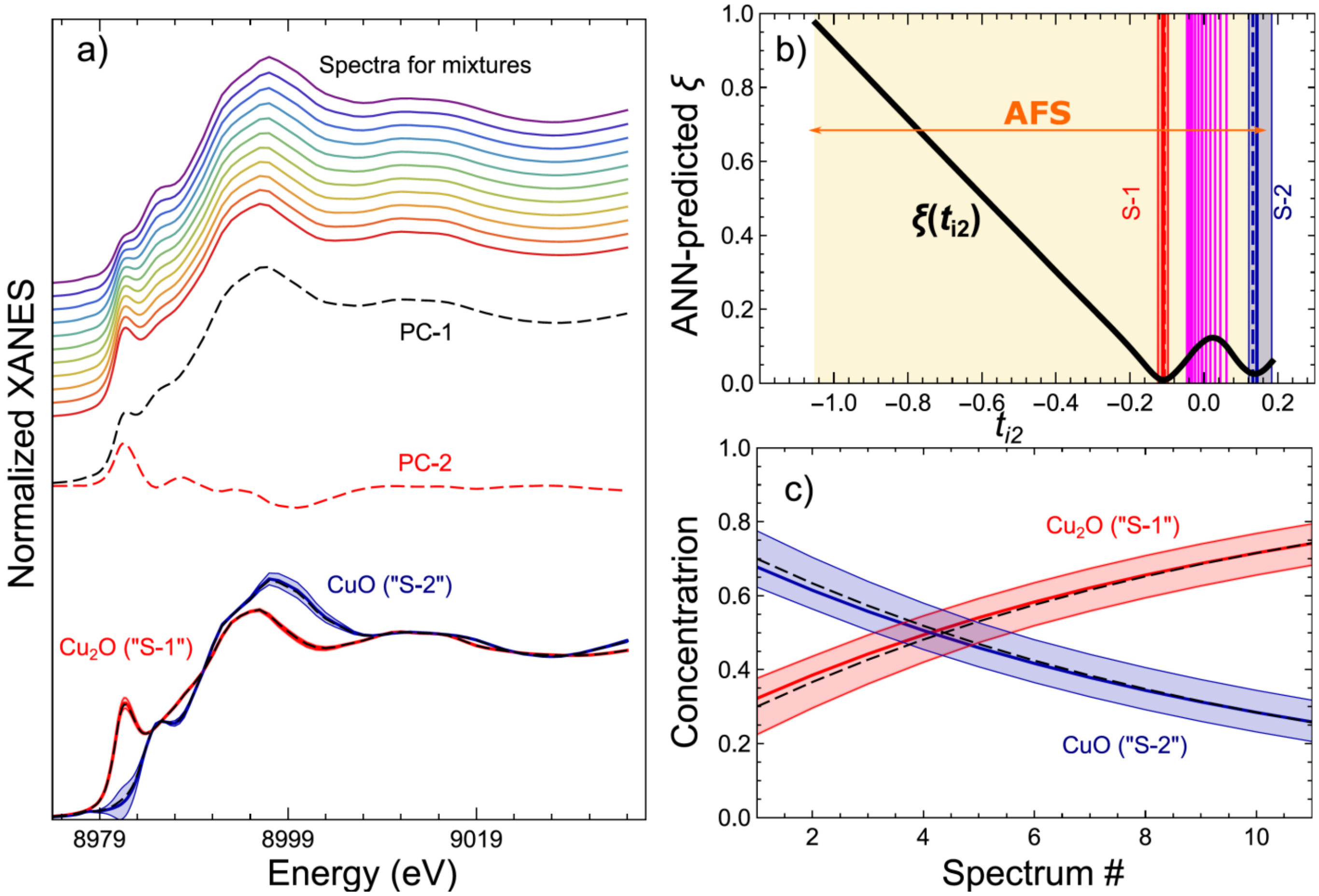

Figure S5. Application of the ANN-MCR method to the model dataset with two species mixture. (a) Model dataset consisting of Cu K-edge XANES spectra, constructed as linear combinations of reference spectra for metallic Cu and CuO reference spectra, using the concentration profiles shown in (c). The true pure spectra and the PCs extracted from the dataset are also shown in (a). All solutions to the MCR problem (candidate solutions for pure spectra) can be represented as $PC_1 + t_{i2} PC_2$. The yellow region in (b) shows the range of t_{i2} values that results in non-negative pure species spectra. Magenta lines in (b) indicate the projections of spectra in the original dataset on the 2nd PC. Any pair of t_{12} and t_{22}, where both values are within the yellow region, t_{12} is to the left of the left-most magenta line, and t_{22} is to the right of the right-most magenta line, will be a valid solution to the MCR problem. This means it satisfies the non-negativity and normalization constraints for the spectra and concentration profiles. From all these possible solutions we can select a single solution, by using ANN_{T+E} networks to obtain the dependency of score ξ on t_{i2} value (black line in (b)), and choosing those t_{i2} values that correspond to the minima of $\xi(t_{i2})$ function (vertical solid red and blue lines in (b)). The shaded blue and red regions in (b) reflect the uncertainty (maximal deviation) of the minima, as obtained by comparing the results obtained with 20 independently trained ANN_{T+E} neural networks. The t_{i2} values corresponding to the true solution are shown with vertical dashed lines in (b). The corresponding spectra and concentration profiles are compared with the true solutions in (a) and (c).

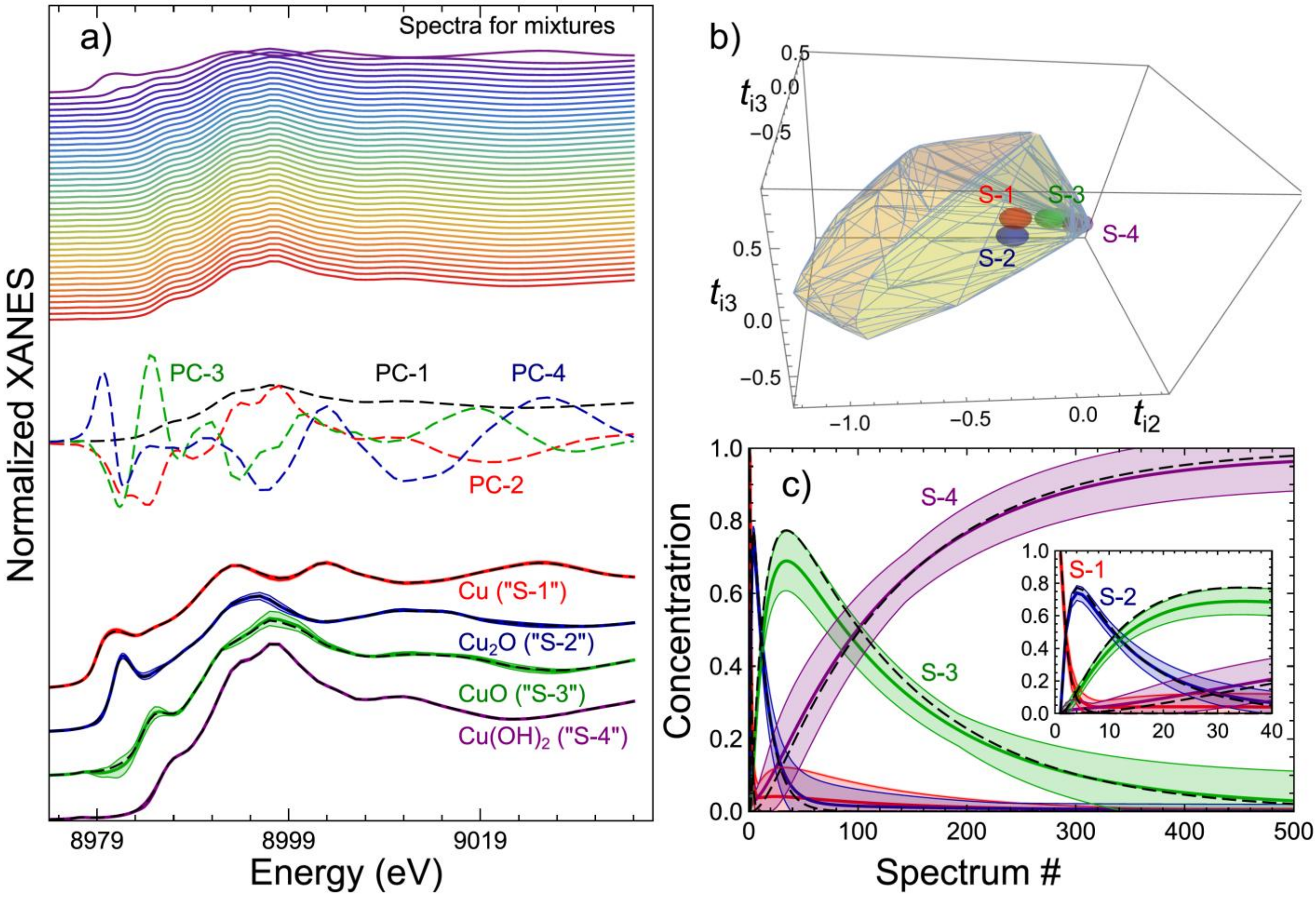

Figure S6. Application of the ANN-MCR method to the model dataset with four species mixture. (a) Model dataset consisting of Cu K-edge XANES spectra, constructed as linear combinations of reference spectra for metallic Cu, Cu_2O, CuO and $Cu(OH)_2$ reference spectra, using the concentration profiles shown in (c). The true pure spectra and the PCs extracted from the dataset are also shown in (a). All solutions to the MCR problem that result in non-negative pure species spectra (spectra-specific AFS) are within the yellow polyhedron in (b). The solutions to the MCR problem yielded by 20 independently trained ANN_{T+E} neural networks correspond to red, green, blue and purple regions in (b). The corresponding spectra and concentration profiles are compared with the true solutions (black dashed lines) in (a) and (c). Inset in (c) shows the zoom-in into the concentration profiles corresponding to the first 40 spectra in the dataset.

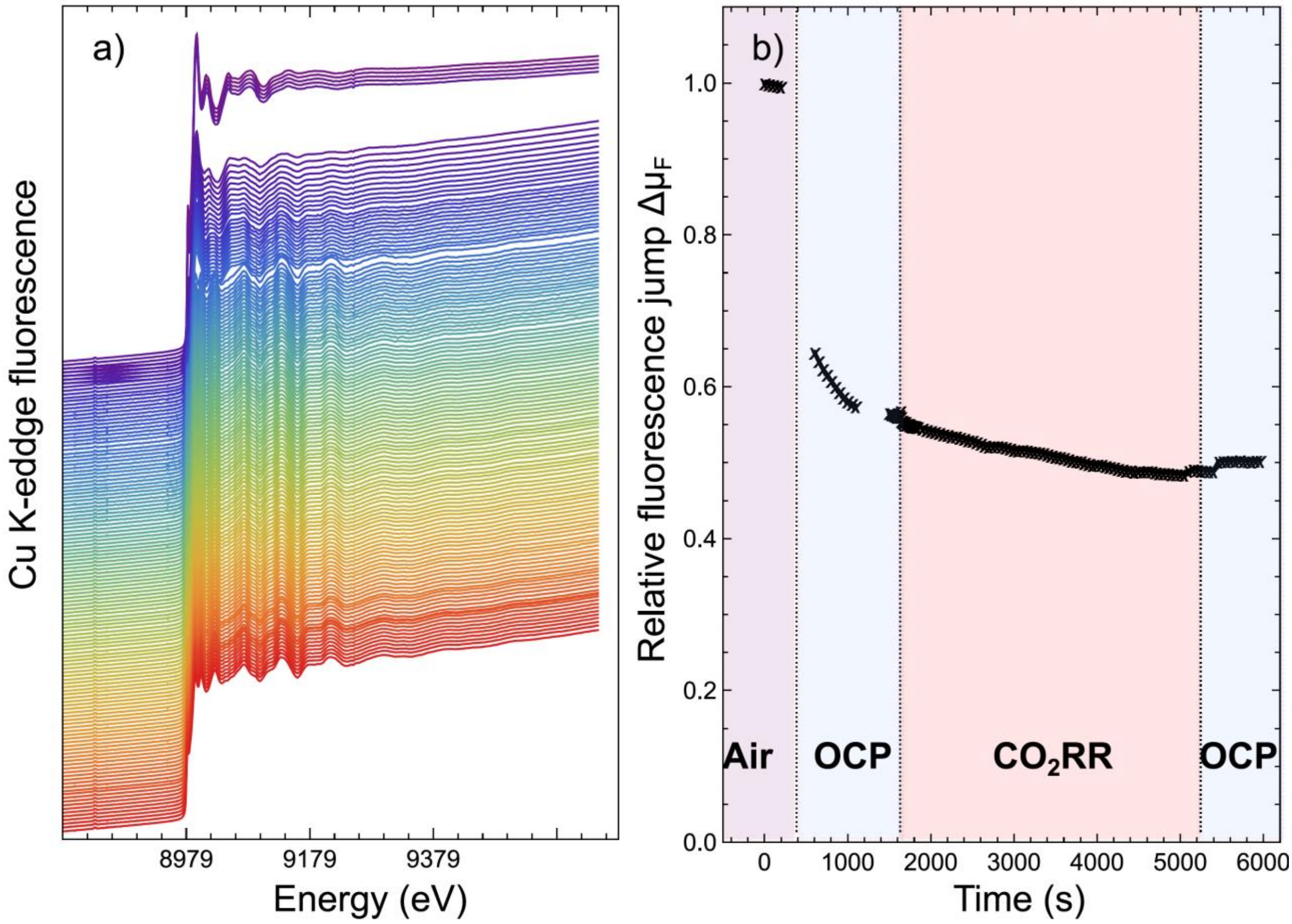

Figure S7. Operando X-ray absorption data for Cu_2O nanocubes (NCs) under acidic CO_2RR conditions. (a) Spectra collected in fluorescence mode in air, in electrolyte (0.1 M oxalic acid and 0.1 M KOH mixture) at open circuit potential, and under CO_2RR conditions (-100 mA/cm^2 current density) and after reaction are shown. Spectra are shifted vertically for clarity, but are not normalized. (b) Corresponding time-dependency of the relative fluorescence signal edge jump at the absorption edge ($\Delta\mu_F$).

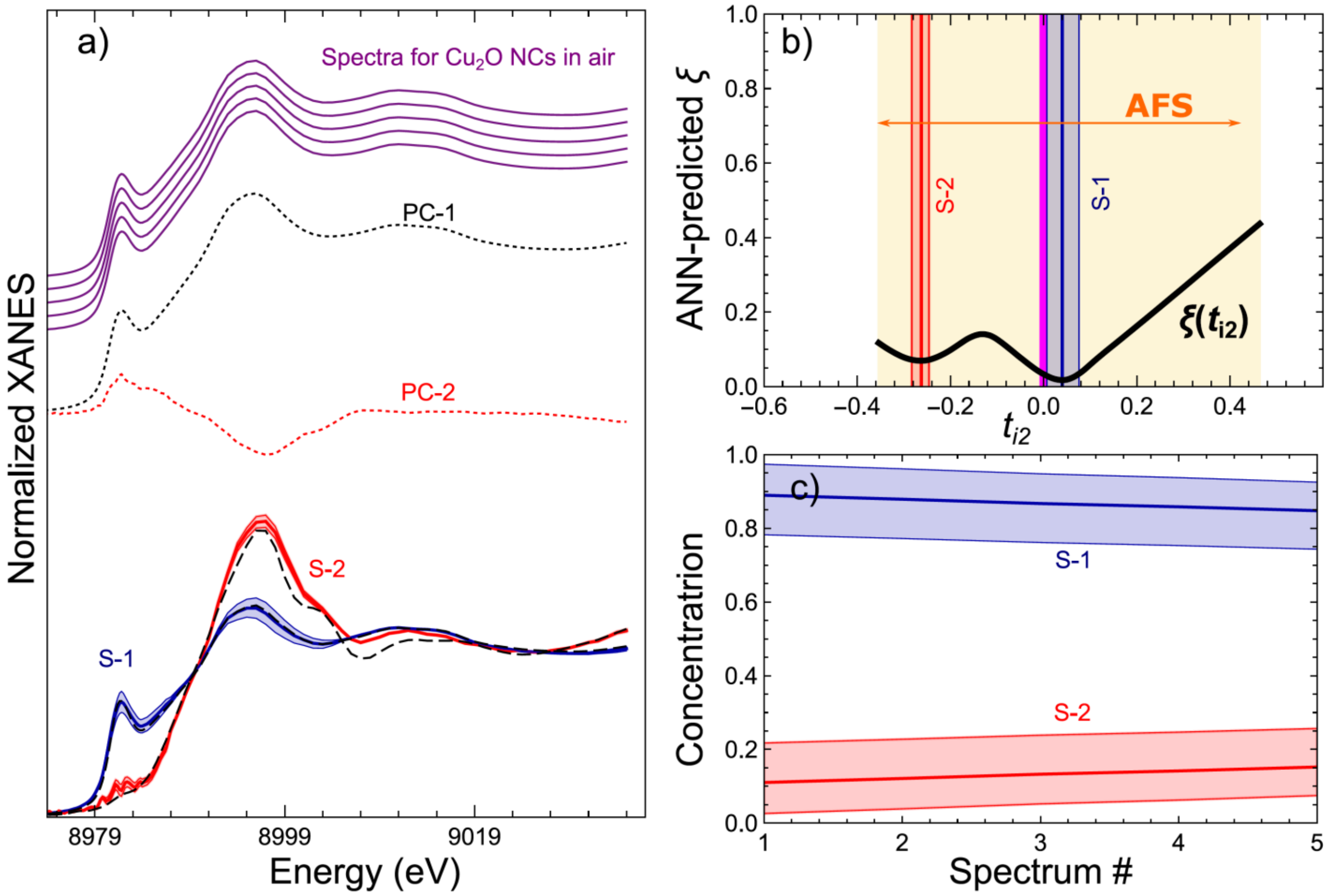

Figure S8. Speciation of Cu_2O NCs in air upon exposure to X-ray beam. (a) Operando time-resolved Cu K-edge XANES spectra for Cu_2O NCs collected in fluorescence mode in air (solid purple lines), first two PCs, extracted from this dataset (dotted black and red lines), and the spectra for species S1 and S2 (solid blue and red lines) that are reconstructed from the PCs by the ANN-MCR method. Here all solutions to the MCR problem (candidate solutions for pure spectra) can be represented as $PC_1 + t_{i2}\,PC_2$. Yellow region in (b) shows the range of t_{i2} values that results in non-negative pure species spectra. Magenta lines in (b) indicate the projections of experimental spectra in the original dataset on the 2nd PC. Any pair of t_{12} and t_{22}, where both values are within the yellow region and t_{12} is to the left of the left-most magenta line, and t_{22} is to the right of the right-most magenta line, will satisfy the non-negativity and normalization constraints for spectra and concentration profiles. From all these possible solutions we can select a single solution, by using ANN_{T+E} networks to obtain the dependency of score ξ on t_{i2} value (black line in (b)), and choosing those t_{i2} values that correspond to minima of $\xi(t_{i2})$ function (vertical solid red and blue lines in (b)). In this case, the contribution of S2 species to the experimental dataset is low. As a result (and as discussed in Figure 6 in the main text), in this case for t_{22} two different minima are obtained: one with t_{22} values close to 0 (i.e., close to the minimum for S1 species, and the second at t_{22} values close to -0.3. The former solution corresponds to the situation where species S1 and S2 are very similar (both similar to Cu_2O reference). The latter solution corresponds to the situation where Cu_2O-like S1 species coexists with small amounts S2 species, where Cu is in 2+ oxidation state. Here we select the second possibility, and for the S2 species always select the minimum that is closer to -0.3 value. The shaded blue and red regions in (b) reflect the uncertainty (maximal deviation) of the minima, as obtained by comparing the results obtained with 20 independently trained ANN_{T+E} neural networks. The corresponding spectra and concentration profiles are shown in (a) and (c), respectively. In (a) the obtained spectra are compared with the best matching experimental reference spectra from our library (black dashed lines). In particular, the spectrum for S1 species matches very well the spectrum for Cu_2O. For the spectrum for S2 species, the spectrum that gives the best match with the spectrum extracted by the ANN-MCR method, is the reference spectrum for Cu_2FeO_4. This indicates that in S2 species Cu(II) species are forming oxide phase with distorted octahedral coordination. However, due to the very low contribution of these species to our dataset, unambiguous identification of specific phase for these species formed under X-ray irradiation is challenging.

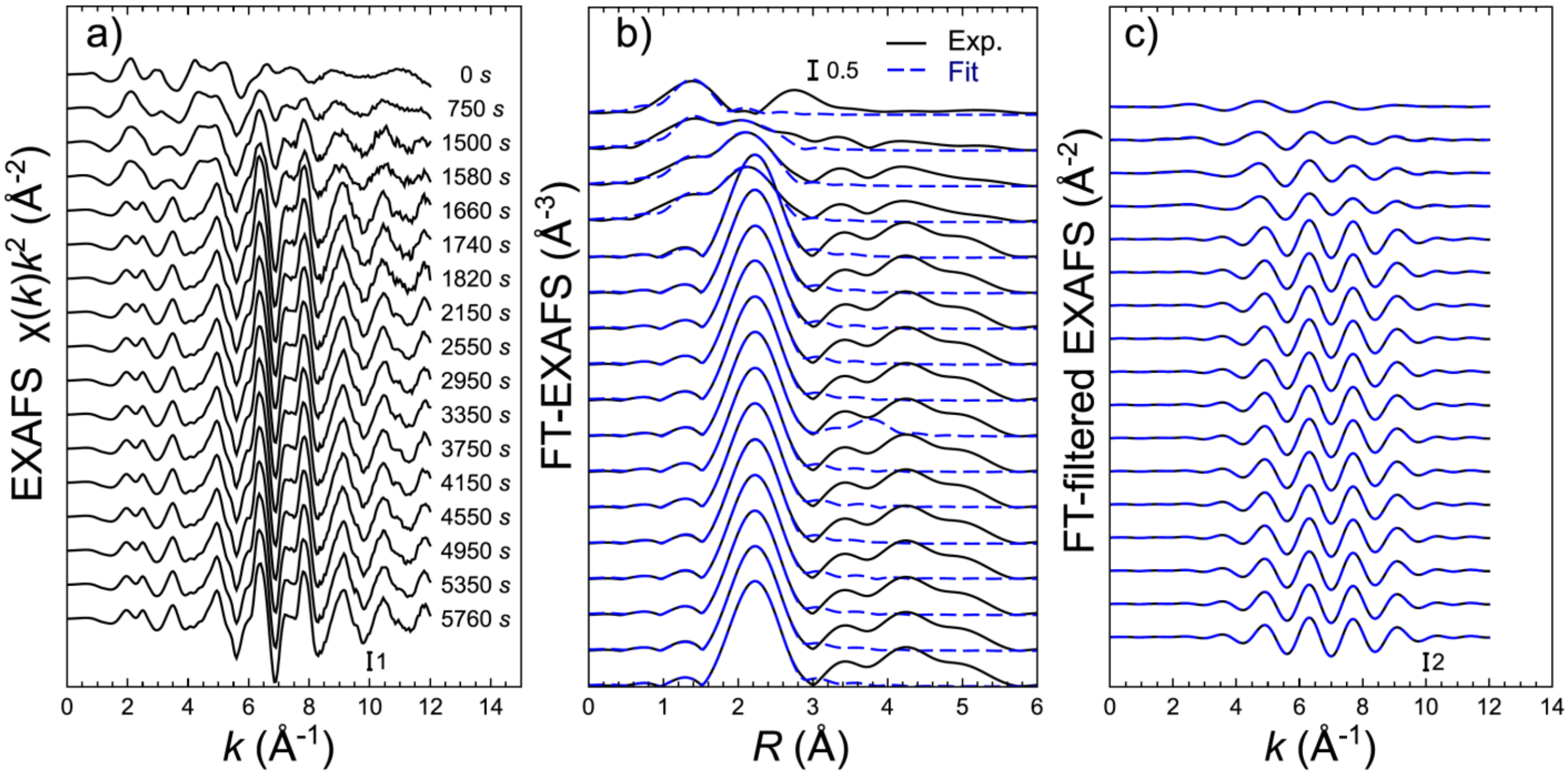

Figure S9. Operando time-dependent EXAFS data for Cu_2O nanocubes (NCs) under acidic CO_2RR conditions. (a) Raw EXAFS spectra in k-space. (b) Corresponding Fourier-transformed (FT) EXAFS spectra (phase uncorrected). (c) FT-filtered EXAFS data. In (b,c) experimental spectra are compared with EXAFS fitting results. Spectra are shifted vertically for clarity.

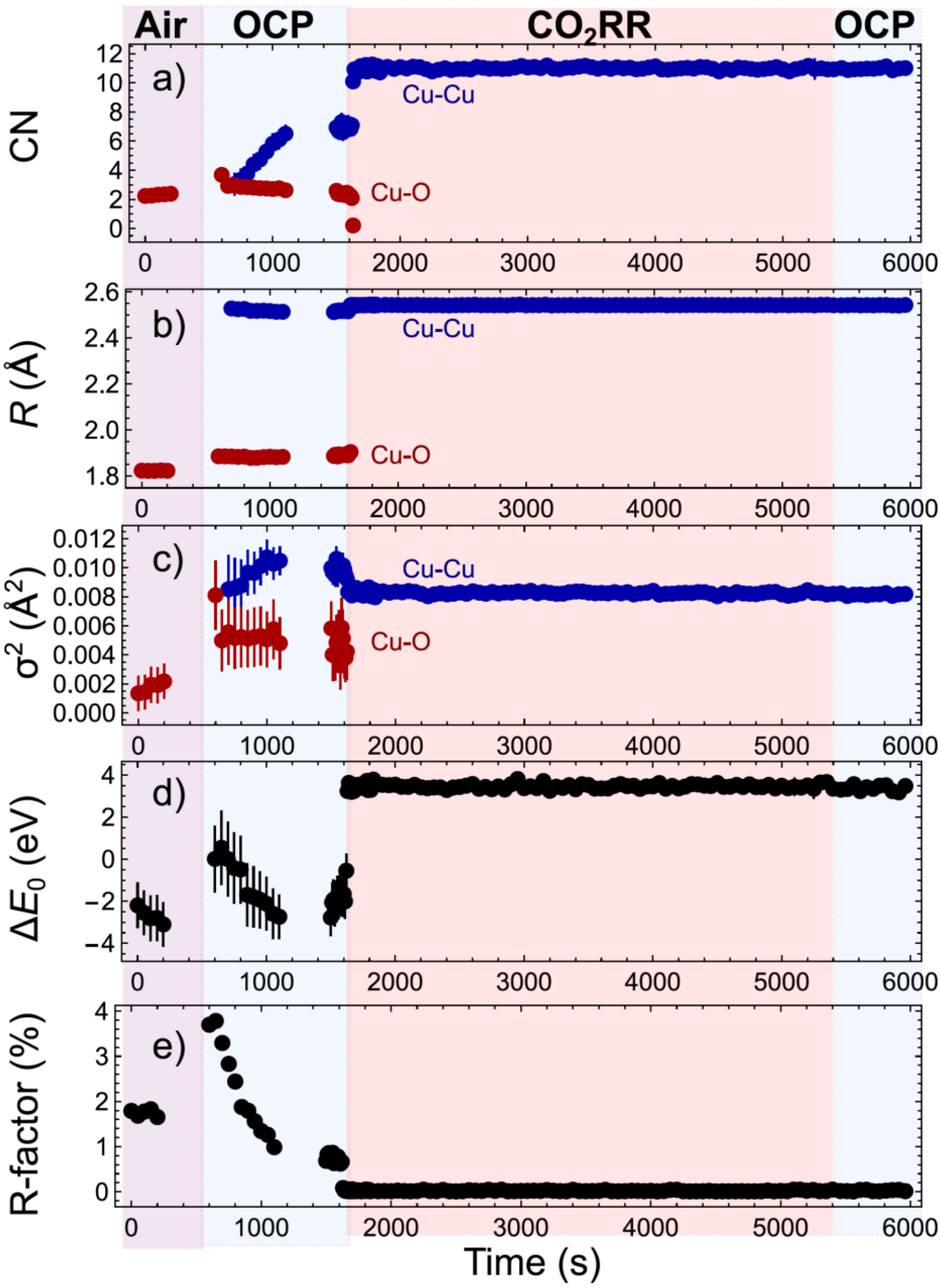

Figure S10. EXAFS fitting results. (a) Cu-Cu and Cu-O coordination numbers (CN). (b) Cu-Cu and Cu-O average interatomic distances (*R*). (c) Cu-Cu and Cu-O disorder (Debye-Waller) factors σ^2. (d) Correction to photoelectron reference energy ΔE_0. (e) Fit R-factors.

Notes and references

1. O. Bunău and Y. Joly, J. Phys.: Condens. Matter, 2009, 21, 345501.
2. A. A. Guda, S. A. Guda, A. Martini, A. Kravtsova, A. Algasov, A. Bugaev, S. P. Kubrin, L. Guda, P. Šot and J. A. van Bokhoven, npj Computational Materials, 2021, 7, 203.
3. J. J. Rehr, J. J. Kas, F. D. Vila, M. P. Prange and K. Jorissen, Phys. Chem. Chem. Phys., 2010, 12, 5503-5513.
4. K. Mathew, C. Zheng, D. Winston, C. Chen, A. Dozier, J. J. Rehr, S. P. Ong and K. A. Persson, Scientific Data, 2018, 5, 180151.
5. C. Zheng, K. Mathew, C. Chen, Y. Chen, H. Tang, A. Dozier, J. J. Kas, F. D. Vila, J. J. Rehr and L. F. Piper, npj Computational Materials, 2018, 4, 12.
6. A. Martini, D. Hursán, J. Timoshenko, E. Ortega, F. Haase, H. S. Jeon, M. Rüscher, C. Rettenmaier, A. Herzog and B. Roldán Cuenya, J. Am. Chem. Soc., 2023, 145, 17351-17366.
7. Wolfram Mathematica 11.3, 2018, Wolfram Research Inc., Champaign, Illinois.
8. G. Klambauer, T. Unterthiner, A. Mayr and S. Hochreiter, Advances in neural information processing systems, 2017, 30.
9. S. Kirkpatrick, C. D. Gelatt and M. P. Vecchi, science, 1983, 220, 671-680.
